# A DNMT3A PWWP mutation leads to methylation of bivalent chromatin and growth retardation in mice

**DOI:** 10.1038/s41467-019-09713-w

**Published:** 2019-04-23

**Authors:** Gintarė Sendžikaitė, Courtney W. Hanna, Kathleen R. Stewart-Morgan, Elena Ivanova, Gavin Kelsey

**Affiliations:** 1 Epigenetics Programme, Babraham Institute, Cambridge, CB22 3AT UK; 2 Centre for Trophoblast Research, University of Cambridge, Cambridge, CB2 3EG UK; 3 Present Address: Biotech Research & Innovation Centre, 2200 Copenhagen, Denmark

**Keywords:** Epigenetic memory, DNA methylation

## Abstract

DNA methyltransferases (DNMTs) deposit DNA methylation, which regulates gene expression and is essential for mammalian development. Histone post-translational modifications modulate the recruitment and activity of DNMTs. The PWWP domains of DNMT3A and DNMT3B are posited to interact with histone 3 lysine 36 trimethylation (H3K36me3); however, the functionality of this interaction for DNMT3A remains untested in vivo. Here we present a mouse model carrying a D329A point mutation in the DNMT3A PWWP domain. The mutation causes dominant postnatal growth retardation. At the molecular level, it results in progressive DNA hypermethylation across domains marked by H3K27me3 and bivalent chromatin, and de-repression of developmental regulatory genes in adult hypothalamus. Evaluation of non-CpG methylation, a marker of de novo methylation, further demonstrates the altered recruitment and activity of DNMT3A^D329A^ at bivalent domains. This work provides key molecular insights into the function of the DNMT3A-PWWP domain and role of DNMT3A in regulating postnatal growth.

## Abstract (web summary)

PWWP domains of DNMT3A and DNMT3B are proposed to interact with H3K36me3. Here the authors present a mouse model carrying a D329A point mutation in the DNMT3A PWWP domain and find this causes dominant postnatal growth retardation, with aberrant progressive gain of DNA methylation across domains marked by H3K27me3 in adult tissues.

## Introduction

DNA methylation is an epigenetic modification that can regulate gene expression by inhibiting transcription factor binding and mediating changes of chromatin conformation during development^1,2^. The inactive X chromosome, imprinted genes, transposons, and repetitive elements are silenced by DNA methylation, together with additional epigenetic modifications. Furthermore, DNA methylation plays a key role in regulating tissue-specific gene expression during development, allowing differential cellular fate determination during lineage specification^3–6^. Methylation is added de novo to DNA at carbon-5 of cytosine bases by DNA methyltransferase (DNMT) 3A, DNMT3B and DNMT3C, although the last is confined to the murine male germline^7–10^. DNA methylation is subsequently maintained by DNMT1, which localises at the replication fork, recognises hemi-methylated DNA and methylates the nascent strand^11–14^. The last member of the DNMT protein family is DNMT3L, a catalytically inactive but essential co-factor for gametic methylation events^15–17^. DNMT3A and DNMT3B show highly conserved structural similarity and partial functional redundancy, but there are biological contexts in which one protein predominates^18–20^. DNMT3B is the major DNMT required for DNA methylation programming during embryogenesis and *Dnmt3b*^*−/−*^ mice die prior to embryonic day 11.5 (E11.5)^18,20^. DNMT3A is predominantly responsible for DNA methylation in oogenesis^21,22^ and neurogenesis^23^, and *Dnmt3a*^*−/−*^ mice fail to survive more than a few weeks after birth^18^. Mutations in human *DNMT3A* are uniquely associated with haematological malignancies and growth abnormalities^24–27^, while *DNMT3B* mutations cause the Immunodeficiency, Centromeric instability, Facial anomalies (ICF) syndrome^28^.

DNMT3A and DNMT3B can methylate cytosines in both CpG and CpH contexts, but show rather limited specificity over flanking sequence composition^29–31^. Thus, post-translational modifications (PTMs) of histone tails are believed to regulate the recruitment of DNMT3s through interactions with their ADD (ATRX-DNMT3-DNMT3L) and PWWP (Pro-Trp-Trp-Pro motif) domains^32^. The ADD domain contains a motif homologous to plant-homeodomain (PHD) zinc fingers, and is shared between DNMT3s and the alpha thalassaemia/mental retardation syndrome X-linked (ATRX) protein^33,34^. The ADD domain of all DNMT3s positively interacts with the amino-terminal tail of histone 3 but is inhibited by histone 3 lysine 4 (H3K4) di-methylation or tri-methylation, marks typically associated with active chromatin^34–37^. The catalytic domain of DNMT3A is autoinhibited by the ADD domain and upon the recognition of unmethylated H3K4 a conformation change unmasks the catalytic site^37,38^.

The PWWP domain is found in DNMT3A, DNMT3B and many histone methyl-transferase or acetyl-transferase proteins^39^. In DNMT3A and DNMT3B, the domain is required for their localisation to major satellite repeats at pericentromeres^40,41^. A mutation in the PWWP domain of DNMT3B was identified in a familial ICF syndrome case, where patients showed loss of DNA methylation at satellite repeat regions^42^. In general, PWWP domains have been shown to interact with methylated histone tails, including but not limited to H3K36me3, a mark found along actively transcribed genes^39,43,44^. Biochemical studies, including chromatin precipitation and peptide arrays, have reproducibly demonstrated that the PWWP domain of DNMT3A interacts exclusively with H3K36me2/3^44–47^. Furthermore, a point mutation (D329A) in a conserved aspartic acid residue in the PWWP domain abrogated the ability of the DNMT3A-PWWP domain to bind H3K36me2 and H3K36me3 peptides or pull down H3K36me2/3-containing native nucleosomes^44–47^. The D329A mutation also reduced the preferential heterochromatin localisation of DNMT3A2 in transfected cells and decreased catalytic activity on unmethylated native nucleosomes^44^. Surprisingly, it was subsequently shown that when assayed in mouse embryonic stem (ES) cells, the PWWP domain of DNMT3B, but not of DNMT3A, was responsible for H3K36me3 recognition and associated gene body methylation, a mechanism that was also successfully modelled in yeast^48,49^. However, whilst mutations in the DNMT3B-PWWP domain or H3K36me3 depletion resulted in loss of gene body methylation in ES cells and yeast^48,49^, in epiblast-like stem cells de novo methylation was reported to occur over transcribed regions in the absence of H3K36me3^50^. Thus, the role of the PWWP domain in targeting de novo DNMTs to chromatin remains enigmatic and, in particular, highlights the necessity for in vivo studies to investigate the mechanisms targeting DNMT3A to chromatin and the function of the DNMT3A PWWP domain.

In this study, we generate mice carrying a point mutation (*Dnmt3a*^*D329A*^) in the DNMT3A-PWWP domain, which unexpectedly is gain of function and manifests phenotypically as dominant postnatal growth retardation. We show that DNMT3A^D329A^ targets H3K27me3 and bivalent chromatin-marked domains for DNA hypermethylation, which is associated with loss of repression of developmental transcription factor genes and altered histone PTM landscape. Thus, a mutation in DNMT3A predicted to abrogate an interaction with H3K36me2/3 causes a complex, gain-of-function phenotype in vivo associated with recruitment of DNMT3A towards H3K27me3-marked chromatin.

## Results

### Generation and phenotypic analysis of *Dnmt3a*^*D329A*^ mice

**Fig. 1:**
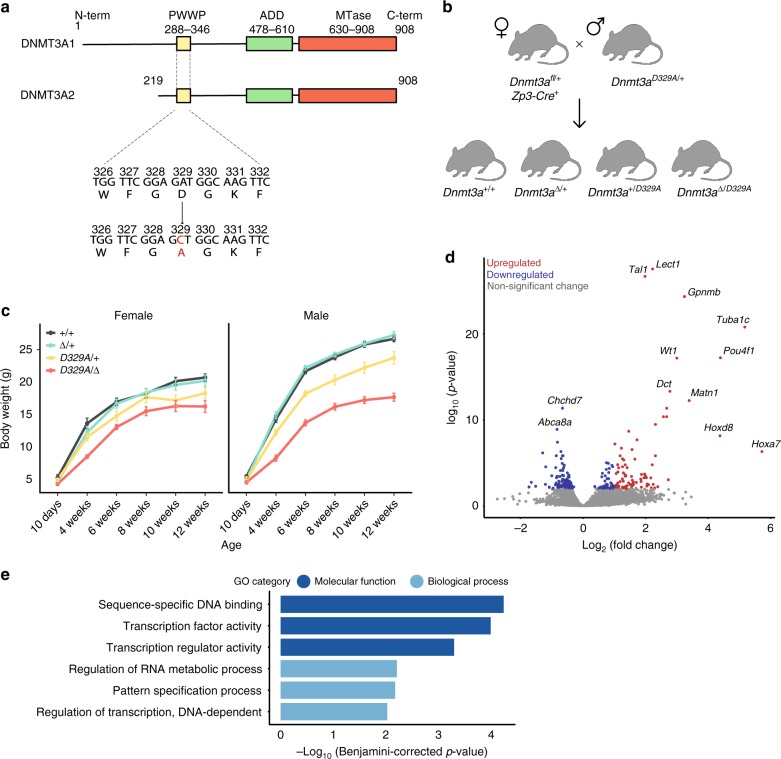
Phenotypic description of mice carrying the *Dnmt3a*^*D329A*^ allele. **a** Schematic representation of the DNMT3A protein isoforms and location of the base substitution, coloured red, introduced to generate the D329A mutation. NCBI IDs: CCDS36397.1, CCDS25784.1. Known domains are indicated by coloured boxes, based on the PROSITE database; numbers indicate amino acid residues. **b** Breeding scheme used to generate offspring of four different genotypes. *+*; wild-type allele; *fl*: *Dnmt3a* containing *loxP* sites surrounding exon 18; *Δ*: *loxP* sites after recombination leading to deletion of exon 18; *Zp3*-*Cre*: oocyte-specific *Zp3* promoter-driven *Cre* recombinase^21,22^; *D329A*: a *Dnmt3a* allele point missense mutation in exon 8. **c** Growth curves indicating change in body weight of male and female mice of different genotypes over the indicated post-natal weeks. Mixed model ANOVA (KR-method) shows overall significant change dependent on genotype and time in both sexes (*p* < 0.001, df(males) = 15,229.48, df(females) = 15,142.15), *F* = 0). Subsequent pairwise Bonferroni-adjusted *t*-test comparisons show that *Dnmt3a*^*Δ/D329A*^ males become significantly underweight at week 4 (*p* < 0.001), and *Dnmt3a*^*+/D329A*^ at week 6 (*p* < 0.001). *Dnmt3a*^*Δ/D392A*^ females become significantly underweight starting from week 4 (*p* = 0.002), with the exception of week 8 (*p* = 0.095). *Dnmt3a*^*+/D329A*^ females do not show significant change. *n* values are given in Supplementary Table 3. Error bars indicate standard error of the mean. Raw data are provided in Source Data. **d** Volcano plot showing gene expression fold change and its significance between *Dnmt3a*^*+/+*^ and *Dnmt3a*^*Δ/D329A*^ adult (14-week) hypothalamus. Differentially expressed genes were determined using DEseq (*p* < 0.01, Benjamini–Hochberg multiple comparisons correction). *n*(*+/+*) = 5, *n(Δ/D392A*) = 4. Selected genes showing the most significant and highest fold change are named. **e** Gene ontology (GO) analysis of differentially expressed genes; only significant terms are shown. Benjamini–Hochberg corrected *p*-value < 0.1 was used as a threshold

To investigate the in vivo significance of the DNMT3A PWWP domain, we generated a mouse carrying the previously in vitro characterised D329A mutation^44–47^ in the endogenous *Dnmt3a* locus (Fig. 1a and Supplementary Fig. 1). Mating *Dnmt3a*^*+/D329A*^ heterozygous males with wild-type females resulted in 19 out of 23 confirmed pregnancies producing a litter, with an average litter size of 8. In contrast, only 2 out of 19 confirmed *Dnmt3a*^*+/D329A*^ female pregnancies produced a litter, with an average litter size of 2 (Supplementary Table 1). Paternal transmission litters showed over 50% post-weaning survival, whilst there were no survivors on maternal transmission. *Dnmt3a*^*+/D329A*^ mothers consistently went overdue and experienced labour dystocia. To assess whether a problem in parturition accounted for the maternal transmission deficit, we performed caesarean section on *Dnmt3a*^*+/D329A*^ females and transferred offspring to foster mothers. This resulted in 45% post-natal litter survival, suggesting that the parturition failure is a dominant effect in females carrying the D329A mutation (Supplementary Table 2), a phenotype that is not explored further in this study.

We proceeded by transmitting the D329A mutation from males crossed to females with heterozygous oocyte-specific conditional ablation of *Dnmt3a* (*Δ*)^21,22^, thus generating four genotypes amongst offspring: +*/+*, *Δ/+*, +*/D329A* and *Δ/D329A* (where the maternal allele is listed first); the last carries the D329A mutation as the only functional *Dnmt3a* allele (Fig. 1b). Notably, *Dnmt3a*^*+/D329A*^ and *Dnmt3a*^*Δ/D329A*^ mice displayed significant postnatal growth retardation, which was more severe in the *Dnmt3a*^*Δ/D329A*^ genotype and particularly pronounced in males (Fig. 1c). In contrast, *Dnmt3a*^*Δ/+*^ offspring were indistinguishable from *Dnmt3a*^*+/+*^, indicating no haploinsufficiency of *Dnmt3a* at a phenotypic level, and showing that the *D329A* allele exhibited a gain of function. We confirmed that the growth deficit of *Dnmt3a*^*+/D329A*^ and *Dnmt3a*^*Δ/D329A*^ mice was not associated with reduced serum growth hormone or insulin-like growth factor 1, major modulators of postnatal growth (Supplementary Fig. 2).

### Transcriptional de-repression in *Dnmt3a*^*Δ/D329A*^ hypothalamus

DNMT3A plays a predominant role in de novo methylation during postnatal neurogenesis^23,51^ and the hypothalamus is involved in regulation of body weight through endocrine function, feeding behaviour and energy homoeostasis^52,53^. We therefore evaluated potential gene dysregulation by performing RNA-seq on adult (14-week) hypothalamus. The *D329A* allele did not cause a statistically significant change in *Dnmt3a* transcript levels, or affect the abundance of other *Dnmt* or *Tet* transcripts (Supplementary Fig. 3). However, we identified 259 differentially expressed genes (DEGs) between *Dnmt3a*^*Δ/D329A*^ and *Dnmt3a*^*+/+*^ (Fig. 1d and Supplementary Data 1). Gene ontology (GO) analysis indicated that upregulated genes were enriched in transcriptional regulators (Fig. 1e and Supplementary Data 2). Although downregulated genes did not show any significant GO term enrichment, amongst them were *Ghsr, Bdnf, Sirt1* and *Cartpt*, key hypothalamic regulators of feeding and energy expenditure^54–57^. More striking, however, was the observation that a number of repressed transcription factor genes, such as *Tal1, Pou4f1, Wt1, Hoxa7* and *Hoxd8*, showed dramatic upregulation in the *Dnmt3a*^*Δ/D329A*^ hypothalamus (Fig. 1d). While growth retardation in the mutants could result from altered expression of the aforementioned metabolic regulators or transcription factors, it is important to highlight that, unusually, the most significant transcriptional changes were observed among genes that are normally deeply repressed in adult tissues.

### The DNMT3A^D329A^ mutant causes aberrant gain of methylation

**Fig. 2:**
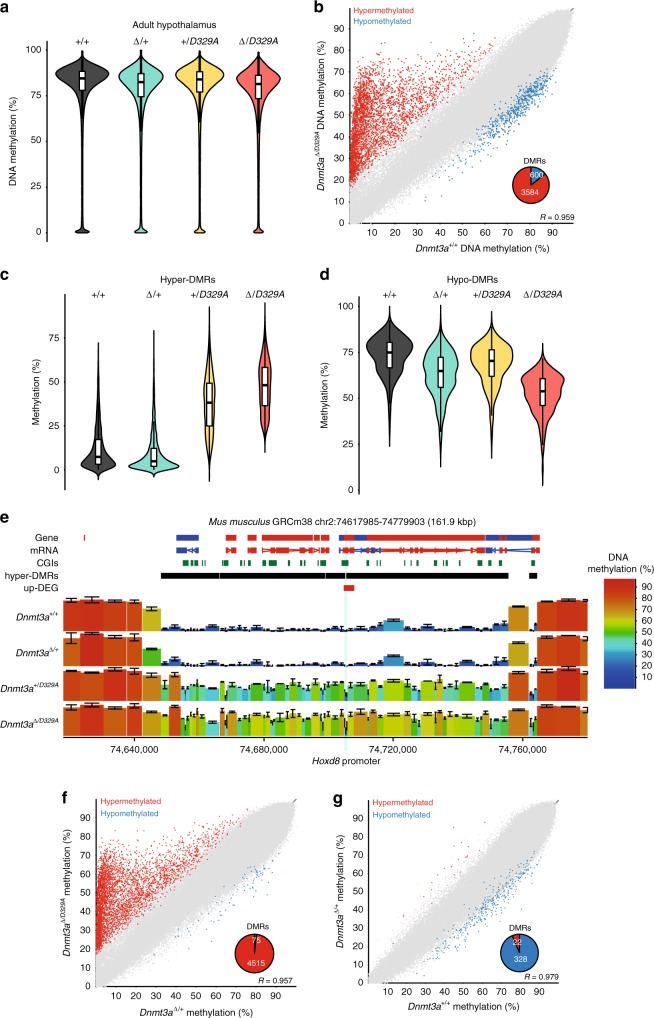
DNA methylation changes in mice carrying *Dnmt3a*^*D329A*^ allele. **a** Beanplots indicating whole genome methylation levels in hypothalamus of adult (14 week, male) mice carrying the alleles shown. Boxplot shows median value and 25–75th percentiles, whiskers show lowest and highest observation, excluding outliers. Raw data are provided in Source Data. **b** Scatterplot showing correlation of methylation levels of individual tiles between *Dnmt3a*^*+/+*^ and *Dnmt3a*^*Δ/D329A*^ mice. Differentially methylated probes were determined using the EdgeR proportion statistic in SeqMonk (*p* < 0.01 corrected for multiple comparisons using Benjamini–Hochberg, methylation difference ≥ 20%). Pie-chart indicates how many of DMRs are hyper-methylated or hypo-methylated in *Dnmt3a*^*Δ/D329A*^. *n* = 3 for each genotype. **c**, **d** Beanplots indicating DNA methylation levels over hypermethylated (**c**) and hypomethylated (**d**) DMRs. Boxplot shows median value and 25–75th percentiles, whiskers show lowest and highest observation, excluding outliers. Source data are listed in Supplementary Data 3. **e** Representative genome browser region showing the *Hoxd* hypermethylated domain. De-repressed gene *Hoxd8* promoter region is shaded. For gene and mRNA tracks, the colour indicates direction, where red is a forward strand and blue is a reverse strand. Colour-coded blocks are tiles of 100-CpG positions. Error bars indicate standard deviation. **f**, **g** Scatterplots showing correlation between methylation levels of 100-CpG tiles in 14-week adult male hypothalamus of **f** *Dnmt3a*^*Δ/+*^ and *Dnmt3a*^*Δ/D329A*^, and **g** *Dnmt3a*^*+/+*^ and *Dnmt3a*^*Δ/+*^. Differentially methylated tiles were determined using the EdgeR proportion statistic in SeqMonk (*p* < 0.01 corrected for multiple comparisons using Benjamini-Hochberg, methylation difference ≥ 20%). The pie-chart indicates the number of DMRs identified. In **a**–**g**, Hypo: hypomethylated, hyper: hypermethylated. Tiles of 100-CpG positions. *n*(*+/+, Δ/+, Δ/D329A*) = 3, *n*(*+/D329A*) = 2. CGI: CpG island, DMR: differentially methylated region

To determine whether changes observed in gene expression were due to aberrant DNA methylation, we performed genome-wide DNA methylation profiling in adult (14-week) hypothalamus. Although the global DNA methylation level was similar across the four genotypes, with mean levels of 77.2%, 75.1%, 77.2%, and 75% in *Dnmt3a*^*+/+*^, *Dnmt3a*^*Δ/+*^, *Dnmt3a*^*+/D329A*^, and *Dnmt3a*^*Δ/D329A*^, respectively, they could be separated by principal component analysis (Fig. 2a, Supplementary Fig. 4a, b). Individual samples clustered together into groups based on genotype, while genotype groups separated spatially, indicating high reproducibility between animals of each genotype, but distinct global methylation patterns between genotypes. The slight reduction in methylation in *Dnmt3a*^*Δ/+*^ in comparison to *Dnmt3a*^*+/+*^ suggested haploinsufficiency at a molecular level. A similar effect was observed between *Dnmt3a*^*+/D329A*^ and *Dnmt3a*^*Δ/D329A*^, suggesting that DNMT3A dosage is important for ~2% of genome-wide DNA methylation levels in this tissue.

As gene bodies are normally marked by H3K36me3, the putative target of the DNMT3A-PWWP domain, we first assessed methylation over gene bodies. All genes were highly methylated irrespective of expression level (Supplementary Fig. 4c), demonstrating that the *Dnmt3a*^*D329A*^ mutation does not profoundly affect gene body DNA methylation, although a similar haploinsufficiency effect to that seen in global methylation was observed in comparing the four genotypes. This effect extended to promoter regions, but for CpG island (CGI) containing promoters the trend was reversed, with higher levels of DNA methylation in the presence of the *Dnmt3a*^*D329A*^ allele (Supplementary Fig. 4d). Surprised by this finding, we used the EdgeR tool to compare *Dnmt3a*^*+/+*^ and *Dnmt3a*^*Δ/D329A*^, which identified 4184 statistically differentially methylated regions (DMRs) (*p*(adj) < 0.01). Strikingly, the majority of DMRs (3584; 85.7%) showed gain of DNA methylation in *Dnmt3a*^*Δ/D329A*^ compared to *Dnmt3a*^*+/+*^ (Fig. 2b–e Supplementary Figs. 5, 6 and Supplementary Data 3). To discriminate the effects of haploinsufficiency versus the *D329A* mutation, and whether hypomethylated domains were a result of the mutation, we used the same statistical tool to identify DMRs between *Dnmt3a*^*Δ/+*^ and *Dnmt3a*^*Δ/D329A*^, and between *Dnmt3a*^*+/+*^ and *Dnmt3a*^*Δ/+*^. In the *Dnmt3a*^*Δ/+*^ and *Dnmt3a*^*Δ/D329A*^ comparison, which corrects for the number of alleles, only 75 out of 4603 DMRs were hypomethylated, whereas in the *Dnmt3a*^*+/+*^ and *Dnmt3a*^*Δ/+*^ comparison, 328 out of 351 DMRs were hypomethylated (Fig. 2f, g and Supplementary Data 4 and 5). This demonstrates that hypomethylation was primarily a result of haploinsufficiency, whilst hypermethylation was caused by the DNMT3A^D329A^ mutation.

Analysis of DNA methylation of DEGs showed that only about one in five misregulated genes were associated with DNA methylation changes (Supplementary Figs. 7–9), suggesting a substantial proportion of DEGs may be attributable to indirect, downstream effects of direct targets. A cluster of genes that showed a gain of DNA methylation over both promoter and gene body stood out in the analysis (Supplementary Fig. 7). Many of these were the same developmental transcription factor genes that are normally strongly repressed in adult hypothalamus, but which are paradoxically derepressed in *Dnmt3a*^*Δ/D329A*^ (e.g., *Hoxa7, Hoxd8, Pou4f1, Tal1*, and *Wt1*). It is important to note that despite the increase in DNA methylation over these genes, their transcription start sites remained relatively less methylated than the surrounding regions (Supplementary Fig. 10).

Taken together, these findings suggest that the *Dnmt3a*^*D329A*^ allele is a driver of aberrant gain of DNA methylation and derepression of a subset of associated genes; consequently, all subsequent analyses are focussed on hypermethylated DMRs.

### DNA methylation alterations occur in multiple tissues

**Fig. 3:**
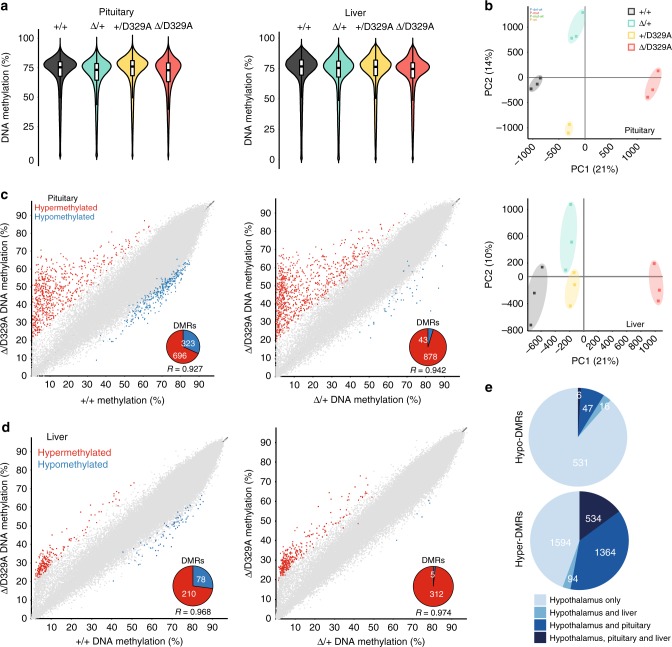
DNA methylation in adult pituitary and liver. **a** Beanplots indicating whole genome methylation levels in pituitary and liver of 14-week adult male mice carrying the alleles shown. Tiles of 300 CpG positions. Boxplot shows median value and 25–75th percentiles, whiskers show lowest and highest observation, excluding outliers. Raw data are provided in Source Data. **b** PCA plots showing clustering of individual pituitary and liver samples into separate distant groups based on the genotype. **c**, **d** Scatterplots showing correlation between methylation levels of individual 300-CpG tiles in 14-week adult male (**c**) pituitary and (**d**) liver between *Dnmt3a*^*+/+*^ and *Dnmt3a*^*Δ/D329A*^, and between *Dnmt3a*^*Δ/+*^ and *Dnmt3a*^*Δ/D329A*^. Differentially methylated tiles were determined using the EdgeR proportion statistic in SeqMonk (*p* < 0.01 corrected for multiple comparisons using Benjamini–Hochberg, methylation difference ≥ 20%). Pie-chart insets indicate how many of DMRs are hyper-methylated or hypo-methylated. **e** Pie charts showing how many hypo-methylated and hyper-methylated tiles in hypothalamus overlap the corresponding differentially methylated tiles in pituitary, liver or both. Pituitary and liver: tiles of 300-CpG position; hypothalamus: tiles of 100-CpG positions. In **a**–**d**, *n*(pituitary, +*/+*, *Δ/+, Δ/D329A*) = 3, *n*(pituitary, +*/D329A*) = 2, *n*(liver) = 3

To assess whether the observed DNA methylation abnormalities were tissue-specific, we also analysed DNA methylation in adult (14-week) pituitary and liver. We chose pituitary because of its functional link with hypothalamus in growth regulation^58^ and liver as an unrelated and relatively homogeneous tissue. As with hypothalamus, the four *Dnmt3a* genotypes had similar global DNA methylation levels in these tissues, but could be separated by principal component analysis (Fig. 3a, b). Using the EdgeR tool to identify DMRs, both liver and pituitary showed an excess of hypermethylated sites similar to, although to a lesser extent than, the hypothalamus (Supplementary Data 6–9). Moreover, hypermethylation in both tissues was specifically related to the presence of the *D329A* allele (Fig. 3c, d). Strikingly, there was substantial overlap of DMRs between the three tissues, particularly amongst hypermethylated probes (Fig. 3e). The occurrence of shared hypermethylated DMRs, albeit with tissue-specific differences in magnitude, raised the question of whether aberrant DNA methylation arose early in embryogenesis prior to cell lineage specification or independently in each tissue.

### DNA methylation gain is progressive in postnatal development

**Fig. 4:**
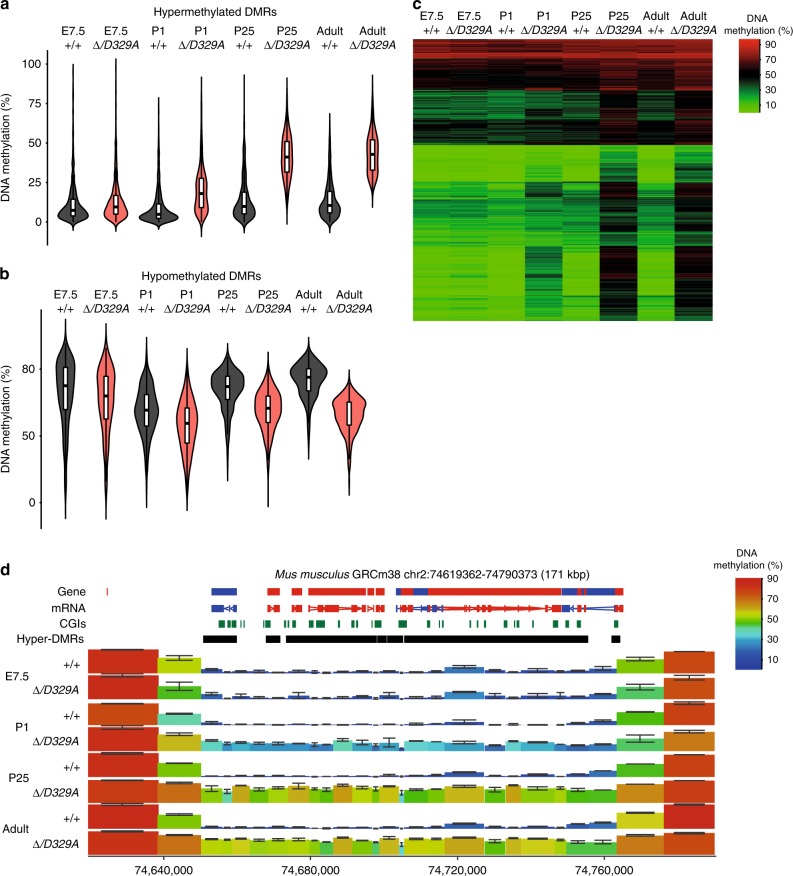
DNA methylation dynamics during development. **a**, **b** Beanplots indicating DNA methylation levels over hyper-methylated (**a**) and hypo-methylated (**b**) DMRs across development in *Dnmt3a*^*+/+*^ and *Dnmt3a*^*Δ/D329A*^ mice. Tiles were quantified over adult hypothalamus DMRs, which were merged if the distance was <1 kb. Boxplots show median value and 25–75th percentiles, whiskers show lowest and highest observation, excluding outliers. Raw data are provided in Source Data. **c** Heatmap showing how methylation is gained at DMRs over time. Shown are 1915 300-CpG tiles, overlapping DMRs identified between *Dnmt3a*^*+/+*^ and *Dnmt3a*^*Δ/D329A*^ in adult (14-week) hypothalamus. DMRs are clustered based on Euclidean method, on the basis of smallest absolute difference between quantitation values. Tiles of 300-CpGs. **d** Genome browser view of the *Hoxd* gene cluster indicating progressive gain in DNA methylation across the whole domain. For gene and mRNA tracks, the colour indicates direction, where red is a forward strand and blue is a reverse strand. CGI: CpG island, hyper-DMR: hypermethylated region identified in adult hypothalamus. Colour-coded blocks indicate tiles of 300-CpG positions. Error bars indicate standard deviation. In **a**–**d**, E7.5 is epiblast, and P1, P25 and adult are hypothalamus. *n*(E7.5) = 4, *n*(P1, P25) = 2, *n*(adult) = 3

The major phase of DNA methylation establishment in mice occurs during early post-implantation development (E4.5–E6.5)^5,59^. To address the possibility that aberrant methylation was established during this phase of embryonic remethylation, we collected E7.5 mouse epiblasts (Epi) from embryos of all four genotypes. Global methylation was more variable in *Dnmt3a*^*+/D329A*^ and *Dnmt3a*^*Δ/D329A*^ embryos than *Dnmt3a*^*+/+*^ and *Dnmt3a*^*Δ/+*^ (Supplementary Fig. 11). Overall, methylation in *Dnmt3a*^*+/D329A*^ and *Dnmt3a*^*Δ/D329A*^ embryos was lower than wild-type at all genomic features; however, at CGI promoters this may be attributable to a haploinsufficiency effect as reduced DNA methylation was also observed in *Dnmt3a*^*Δ/+*^ embryos. We used EdgeR to assess *Dnmt3a*^*+/+*^ and *Dnmt3a*^*Δ/+*^ embryos and identified 136 DMRs out of a total of 108,212 tiles (0.13%; Supplementary Data 10). All but two DMRs were overlapping a CGI, again pointing to the potential role of DNMT3A in CGI methylation during early development. Comparison between *Dnmt3a*^*+/+*^ and *Dnmt3a*^*Δ/D329A*^ embryos identified only five significantly hypomethylated tiles out of a total of 108,212 (0.005%; Supplementary Data 11). This suggests that the presence of mutant DNMT3A results in stochastic delay in de novo methylation across genomic regions rather than any locus specificity. In agreement with this, embryos did not cluster based on genotype (Supplementary Fig. 11b–d). Crucially, there was no evidence of hypermethylation in *Dnmt3a*^*Δ/D329A*^ mutants at E7.5 (Supplementary Fig. 11e–j), suggesting that it was not set during the major wave of post-implantation de novo methylation. In view of this result, and because hypothalamic neurons undergo further epigenetic modelling during postnatal maturation^51^, we next assessed methylation in hypothalamus at postnatal days 1 and 25 (P1 and P25). This showed a progressive gain of DNA methylation across the domains destined to be hypermethylated in *Dnmt3a*^*Δ/D329A*^ adults (Fig. 4a–d). Together, these findings suggest that the aberrant gain of DNA methylation caused by the *D329A* allele occurs in tissues independently during postnatal development rather than early development.

### Hypermethylated loci are enriched in transcription factors

**Fig. 5:**
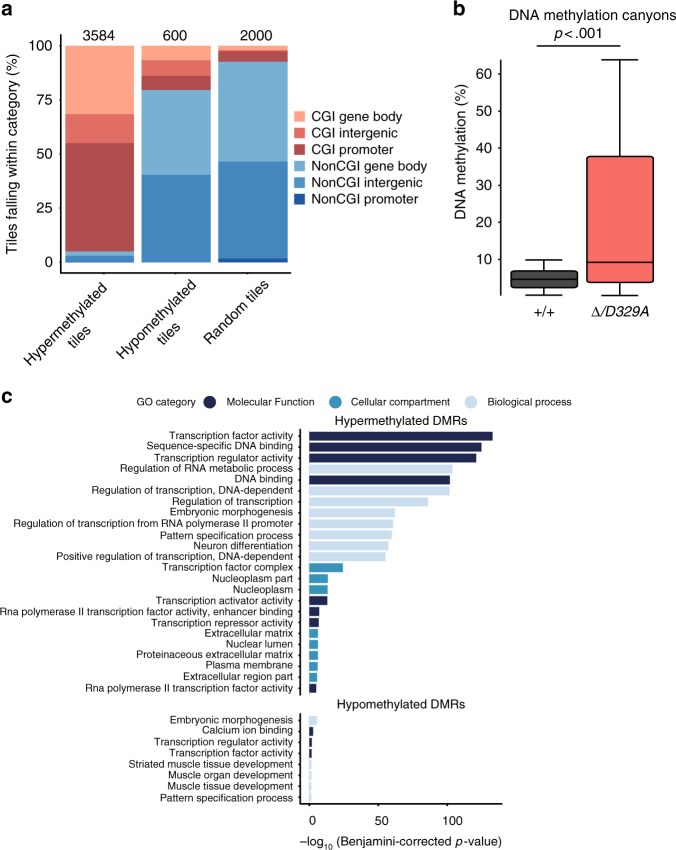
Features of regions aberrantly methylated by DNMT3A^D329A^. **a** Percentages of hypermethylated and hypomethylated tiles (100-CpG) falling within the genomic features indicated compared with a random set of 100-CpG tiles (Supplementary Data 14). *n* (tiles) indicated on top of the bars. CGI: CpG island. **b** Distribution of methylation values over DNA methylation canyon probes^60^ in *Dnmt3a*^*+/+*^ and *Dnmt3a*^*Δ/D329A*^ mice. *n* = 779, Mann–Whitney test (*p* < 0.01, *U*_A_ = 432,773, *z* = −14.57). Boxplots show median value and 25–75th percentiles, whiskers show lowest and highest observation, excluding outliers. Source data are provided in Supplementary Data 12. **c** GO analysis of genes overlapping differentially methylated tiles. Unless there were fewer significant terms, the top 8 most significant terms per category are shown. Benjamini–Hochberg corrected *p*-value < 0.01 was used as a threshold. DMR differentially methylated region

To identify what was shared amongst loci anomalously methylated by DNMT3A^D329A^, we evaluated their genomic properties. As previously observed, hypermethylated DMRs were strongly enriched in CGIs (Fig. 5a). We also noticed that many hypermethylated DMRs fell within large normally unmethylated genomic regions (Fig. 2e), features previously described as methylation ‘valleys’ or ‘canyons’ that are conserved amongst vertebrates^60,61^. Of the 1104 canyons originally defined in hematopoietic stem cells^60^, 779 were also hypomethylated in wild-type hypothalamus (Supplementary Data 12) and displayed a substantial increase in DNA methylation in *Dnmt3a*^*Δ/D329A*^ (Fig. 5b). GO analysis indicated that genes within hypermethylated DMRs in *Dnmt3a*^*Δ/D329A*^ hypothalamus were strongly enriched in transcription factors and regulators of morphogenesis and differentiation (Fig. 5c and Supplementary Data 13).

### Gain of DNA methylation is linked to H3K27me3

**Fig. 6:**
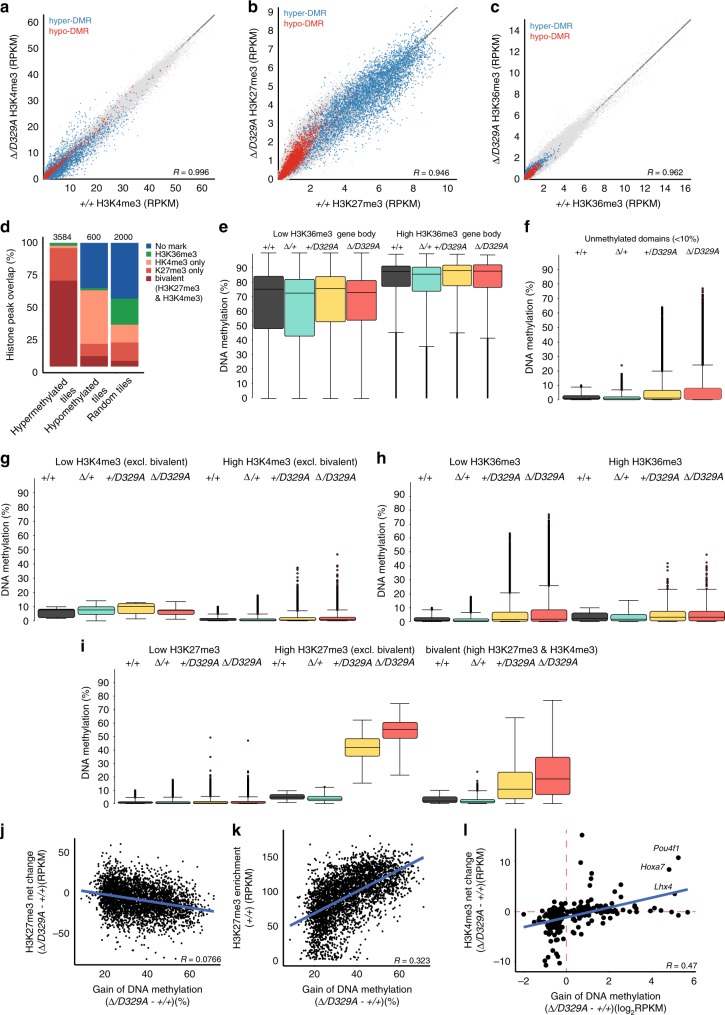
Link between H3 post-translational modifications and aberrant DNA methylation. **a**–**c** Scatterplots showing correlation between enrichments for **a** H3K4me3; **b** H3K27me3; **c** H3K36me3 between *Dnmt3a*^*+/+*^ and *Dnmt3a*^*Δ/D329A*^ hypothalamus, with the tiles overlapping hypermethylated (blue) and hypomethylated (red) DMRs indicated. *n* = 3 each genotype. Tiles of 2 kb with a step of 1 kb were used for analyses. **d** Percentages of hypermethylated and hypomethylated DMRs (100-CpG) that overlap bivalent (H3K4me3 and H3K27me3), H3K27me3 only, H3K4me3 only and H3K36me3 peaks, or regions with no mark. A random set of 100-CpG tiles (excluding DMRs) was used as a whole genome representative (Supplementary Data 14). *n* (tiles) indicated above each bar. H3K36me3 tiles that show overlap with H3K4me3 or H3K27me3 were included in the H3K36me3 group. Peaks were called using MACS peak caller in adult hypothalamus ChIP-seq. **e** DNA methylation levels over gene bodies marked by low or high levels of H3K36me3. DNA methylation quantified over individual gene bodies, excluding promoters. **f** DNA methylation levels across unmethylated genomic regions (<10% DNA methylation in wild-type hypothalamus) amongst different genotypes. **g**, **h** DNA methylation levels between genotypes over unmethylated regions overlapping low and high **g** H3K36me3 or **h** H3K4me3 marked chromatin. **i** DNA methylation levels between genotypes over unmethylated regions overlapping low, high H3K27me3, or both high H3K4me3 and high H3K27me3 (bivalent), marked chromatin. **j** Scatterplot showing the relationship between gain of DNA methylation in *Dnmt3a*^*Δ/D329A*^ hypothalamus and change in enrichment of H3K27me3. Based on H3K27me3 enrichment difference over DMR tiles between *Dnmt3a*^*+/+*^ and *Dnmt3a* ^*Δ/D329A*^ adult hypothalamus. **k** Scatterplot showing relationship between gain of methylation in *Dnmt3a*^*Δ/D329A*^ and initial levels of H3K27me3 enrichment. **l** Scatterplot showing relationship between change in gene expression and change in respective gene promoter H3K4me3 enrichment between *Dnmt3a*^*+/+*^ and *Dnmt3a* ^*Δ/D329A*^ adult hypothalamus. H3K4me3 enrichment was quantitated over gene promoters. In **e**, **g**, **h**, **i** Low level: bottom quintile (0–20%) of enrichment;high level: top quintile (80–100%) of enrichment. In **e**–**i**, boxplots show median value and 25–75th percentiles, whiskers show lowest and highest observation, excluding outliers marked by individual points. Raw data are provided in Source Data. In **g**–**i**, histone mark enrichments were quantitated over 100-CpG DMR tiles

Developmental transcription factor genes that showed dysregulated expression and DNA methylation are embedded in methylation canyons typically marked by bivalent chromatin and silenced by Polycomb repressor complexes^60,62,63^. Thus, we hypothesised that DNMT3A^D329A^ hypermethylated DMRs would be enriched in H3K4me3 and H3K27me3. To assess this, we generated H3K4me3, H3K27me3, and H3K36me3 chromatin immunoprecipitation-sequencing (ChIP-seq) datasets from adult (14-week) hypothalamus. Overall, these histone marks exhibited strong correlation between *Dnmt3a*^*+/+*^ and *Dnmt3a*^*Δ/D329A*^ samples (Supplementary Fig. 12), indicating no marked shift in the genomic distribution of these marks in the presence of the *Dnmt3a*^*D329A*^ allele. Strikingly, however, whereas hypermethylated and hypomethylated DMRs were not obviously separated in terms of enrichment levels for H3K4me3 or H3K36me3, there was a clear separation in their enrichment for H3K27me3 (Fig. 6a–c).

To explore this effect more rigorously, we called H3K36me3, H3K27me3, and H3K4me3 peaks and observed that 70.4% of hypermethylated DMRs were bivalent, i.e., contained both H3K27me3 and H3K4me3 peaks, with an additional 26.3% containing H3K27me3 only peaks (Fig. 6d), suggesting that DNMT3A^D329A^ is preferentially recruited to H3K27me3-marked chromatin and is able to methylate bivalent DNA marked by H3K4me3 in the presence of H3K27me3. In contrast, only 11 (1.8%) hypomethylated and 74 (2.1%) hypermethylated domains overlapped H3K36me3 peaks, again indicating that DMRs were not associated with this histone mark. We then sought to discriminate between two possible mechanisms to explain the aberrant targeting of DNMT3A^D329A^ to Polycomb-associated methylation canyons: (1) the D329A mutation leads to promiscuous localisation and activity of DNMT3A due to a loss of association with H3K36me3-marked chromatin; or (2) DNMT3A^D329A^ is selectively recruited to H3K27me3-marked chromatin. To do this, we looked first at DNA methylation at gene bodies with low (bottom quintile) or high (top quintile) levels of H3K36me3. In a comparison of the four genotypes, we observed no significant differences in gene body DNA methylation associated with the *Dnmt3a*^*D329A*^ allele (Fig. 6e). We then selected regions that are hypomethylated in *Dnmt3a*^*+/+*^ hypothalamus (methylation level below 10%; 4.6% of the genome; Fig. 6f) and partitioned hypomethylated probes based on low or high levels of enrichment of H3K4me3, H3K27me3, or H3K36me3. By this analysis, neither the presence nor absence of either H3K4me3 or H3K36me3 were associated with DNA hypermethylation by DNMT3A^D329A^ (Fig. 6g, h). In contrast, hypomethylated regions highly enriched in H3K27me3 were preferentially hypermethylated (Fig. 6i). This suggests that hypomethylation of genomic domains per se is not sufficient to recruit and activate DNMT3A^D329A^. Furthermore, bivalent regions marked by both H3K27me3 and H3K4me3 also showed a substantial gain in DNA methylation (Fig. 6i), although to a lesser degree than H3K27me3 alone, suggesting the presence of H3K4me3 remains antagonistic to DNMT3A^D329A^ activity. Together these results support the conclusion that DNMT3A^D329A^ is selectively recruited to H3K27me3-marked chromatin.

**Fig. 7:**
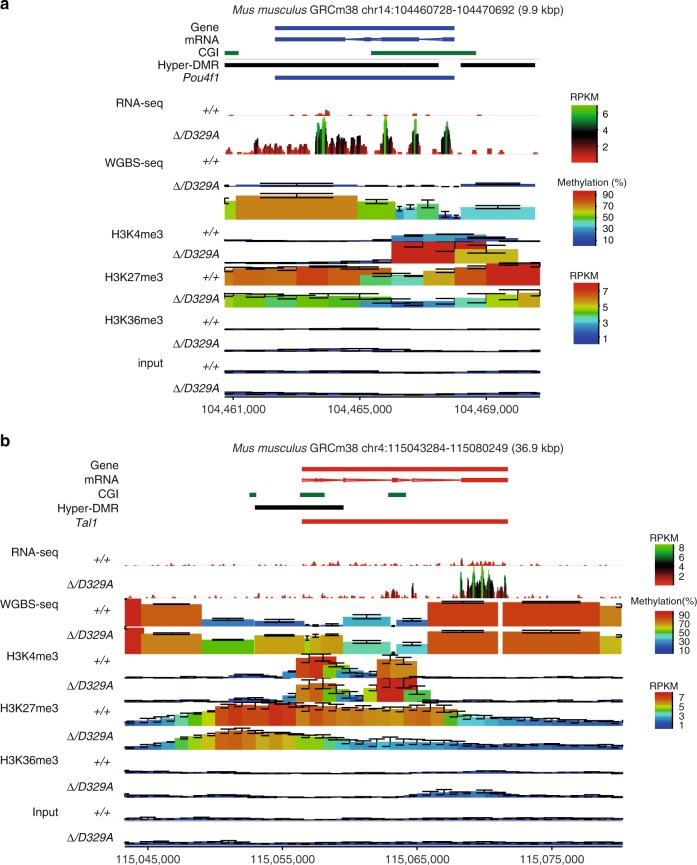
Genomic landscape of de-repressed genes in *Dnmt3a* ^*Δ/D329A*^ hypothalamus. **a**, **b** Representative genome browser views of gene expression, DNA methylation, and distribution of H3K4me3, H3K27me3, and H3K36me3 marks (determined by ChIP-seq) in adult (14-week) male hypothalamus of *Dnmt3a*^*+/+*^ and *Dnmt3a*^*Δ/D329A*^ genotypes. H3K4me3 and H3K36me3 are enriched over actively transcribed CGI promoters and gene bodies, respectively. H3K27me3 shows a broad enrichment over transcriptionally silent genes and loss of enrichment upon methylation gain. Genes **a** *Pou4f1* and **b** *Tal1* are up-regulated and hypermethylated in *Dnmt3a*^*Δ/D329A*^. CGI: CpG island, DMR: hypermethylated region. Each colour-coded block represents: a 50 bp window with 10 bp step size in the gene expression dataset; a 100-CpG window in the methylation dataset; and 2 kb window with 1 kb step size in the histone enrichment datasets. For gene and mRNA tracks, the colour indicates direction, where red is a forward strand and blue is a reverse strand. Error bars indicate standard deviation. *n*(*+/+*) = 5, *n(Δ/D392A*) = 4 for RNA-seq datasets; *n* = 3 for each genotype in WGBS-seq and ChIP-seq datasets

Because H3K27me3 and DNA methylation show mutual exclusivity in some genomic contexts^64–66^, we were interested whether the histone landscape would be altered, with DNA methylation replacing H3K27me3 at hypermethylated DMRs. Although the levels of DNA methylation gain were marginally associated with loss of H3K27me3 (Fig. 6j), these marks appeared to co-exist with hypermethylated domains retaining a substantial amount of H3K27me3, even at CGIs, and we observed that the extent of DNA methylation gain correlated better with initial enrichment of H3K27me3 over the locus than its loss (Fig. 6k). Finally, we were able to use the ChIP-seq data to evaluate that for a subset of DEGs there were associated changes in promoter H3K4me3 (Fig. 6l). These findings support the link between H3K27me3 and abnormal localisation of DNMT3A^D329A^, and suggest that the normal histone landscape is affected by the aberrant gain in DNA methylation (Fig. 7).

### Non-CpG methylation shows altered DNMT3A^D329A^ recruitment

**Fig. 8:**
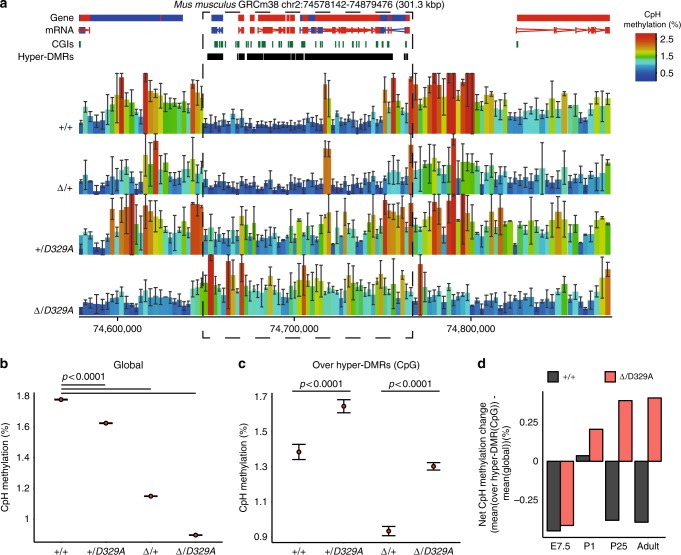
CpH methylation in hypothalamus of *Dnmt3a*^*+/+*^ and *Dnmt3a*^*Δ/D329A*^ mice. **a** Representative genome browser view of CpH methylation in adult (14-week) male hypothalamus. Methylation levels appear to be dependent on the number of alleles present, with a decrease observed in *Dnmt3a*^*∆/+*^ compared to *Dnmt3a*^*+/+*^. Notably, there is an increase of methylation over hyper-DMR regions (highlighted by the box) in the presence of *Dnmt3a*^*D329A*^. Each bar represents a 1000-CpH tile. For gene and mRNA tracks, the colour indicates direction, where red is a forward strand and blue is a reverse strand. CGI: CpG island, hyper-DMR: hypermethylated region. *n*(*+/+, Δ/+, Δ/D329A*) = 3, *n*(*+/D329A*) = 2. Error bars indicate standard deviation. **b** Global mean CpH methylation values across different genotypes. **c** Mean CpH methylation values over DMRs with CpG hypermethylation. **b**, **c** 1000-CpH tile quantitation for chromosomes 2 and 11 was used as representative. Error bars indicate standard error of the mean. Pairwise comparisons were done using a two-tailed *t*-test. Raw data are provided in Source Data. **d** Barplot indicating the net difference in mean CpH methylation between hyper-DMRs and global levels for *Dnmt3a*^*+/+*^ and *Dnmt3a* ^*Δ/D329A*^ mice across development (E7.5 epiblast, P1, P25, and adult hypothalamus). Raw data are provided in Source Data

Non-CpG (CpH) methylation becomes particularly abundant in neural tissues, where it is established postnatally by DNMT3A^67–69^. Importantly, CpH methylation is not maintained at DNA replication, so its presence is a record of de novo methylation since the last replication event. Thus, we investigated CpH methylation in adult hypothalamus as a read-out of DNMT3A localisation and activity (Fig. 8a). CpH methylation levels showed strong evidence for haploinsufficiency at the molecular level, as we observed very similar decreases in global levels in *Dnmt3a*^*Δ/+*^ compared to *Dnmt3a*^*+/+*^, and *Dnmt3a*^*Δ/D329A*^ compared to *Dnmt3a*^*+/D329A*^ (Fig. 8a, b). The smaller global reduction between *Dnmt3a*^*+/D329A*^ and *Dnmt3a*^*+/+*^ and between *Dnmt3a*^*Δ/D329A*^ and *Dnmt3a*^*Δ/+*^ suggests some decrease in methyltransferase activity by the mutant protein. Strikingly, however, the pattern over hypermethylated DMRs was reversed, with *Dnmt3a*^*+/D329A*^ and *Dnmt3a*^*Δ/D329A*^ showing increased CpH methylation compared to *Dnmt3a*^*+/+*^ and *Dnmt3a*^*Δ/+*^, respectively (Fig. 8a, c). Comparing relative CpH methylation across prenatal and postnatal development showed that this effect first becomes evident in the P1 hypothalamus (Fig. 8d). In summary, these data demonstrate that recruitment and subsequent activity of the DNMT3A^D329A^ protein is aberrantly localised preferentially to bivalent chromatin in the postnatal brain.

## Discussion

Here we describe a gain-of-function mutation in the PWWP domain of DNMT3A that results in postnatal growth deficiency in mice and, at a molecular level, aberrant DNA methylation of genomic domains harbouring H3K27me3-silenced developmental regulatory genes. We demonstrate that the gain of DNA methylation occurs specifically during postnatal development and is likely to be a consequence of an altered association of the mutant DNMT3A with chromatin. Furthermore, we observed transcriptional de-repression of a subset of the associated genes, suggesting the aberrant localisation of DNA methylation to these genomic regions paradoxically results in their activation.

The DNMT3A-PWWP domain has been described to bind to H3K36me2 and H3K36me3 in vitro, an interaction that was blocked by targeted mutations, including D329A^27,44,47^. In *Dnmt3a*^*D329A*^ E7.5 embryos, we observed a stochastic delay in de novo methylation that was not localised to particular genomic areas. In adult tissues, however, we did not observe a loss of DNA methylation attributable to the D329A mutation and find no evidence that this mutation alters DNA methylation levels at regions enriched for H3K36me3, suggesting the delays observed in embryonic development do not persist. Together, these findings suggest that the function of DNMT3A in vivo is not necessarily reliant on its association with H3K36me2/3, but that the D329A mutant protein may have reduced methylase activity due to an altered association with chromatin or catalytic activity. Subsequently, the delay in de novo methylation may be recovered with time or DNMT3B may be able to compensate during embryogenesis, supported by previous observations that DNMT3A and DNMT3B have partial redundancy in the post-implantation embryo^18,50^.

In addition to an interaction with H3K36me2/3, wild-type DNMT3A has been found to associate specifically with H3K27me3-marked shores of bivalent CGIs^70,71^. In our study, the DNMT3A^D329A^ mutant aberrantly methylates bivalent and H3K27me3-marked genomic domains in postnatal development. This hypermethylation is selective towards polycomb repressive complex (PRC2)-mediated H3K27me3 domains rather than unmethylated regions of the genome in general, supporting an association between the PWWP domain and H3K27me3 in vivo^27^. Nonetheless, direct interaction evidence between DNMTs and H3K27me3 from biochemical experiments is limited^27,44,47,70^, and thus the nature of this association will need to be explored further.

**Fig. 9:**
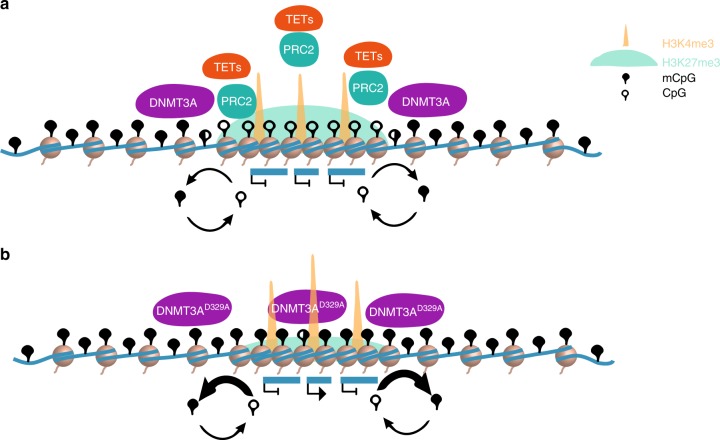
Model of molecular mechanisms acting at bivalent domains. **a** Representation of current understanding of bivalent domain regulation. The PRC2 complex deposits H3K27me3 and compacts chromatin across the promoters and surrounding regions of transcriptionally silent, developmental genes^62^. TET proteins are recruited, which result in active removal of DNA methylation and protection of bivalent chromatin from de novo DNMTs^71^. DNMT3A is recruited to bivalent chromatin shores through an unknown mechanism, enabling active establishment of DNA methylation at the boundaries^60,70,71^. Through the competing actions of TETs and DNMT3A there is a cycle of DNA methylation turnover at the boundaries of bivalent domains. **b** Representation of bivalent chromatin dynamics in the presence of DNMT3A^D329A^. The mutant protein is able to access bivalent chromatin and establish methylation across the whole domain. The presence of DNA methylation appears to influence the local chromatin environment and results in reduction of H3K27me3, potentially due to exclusion of PRC2 from methylated DNA. As a consequence, some genes become de-repressed and show an increase in promoter H3K4me3, while others maintain their transcriptional silencing, this is likely dependent on the availability and strength of necessary transcription factors

DNA methylation and H3K27me3 are repressive marks that have been widely reported to be mutually exclusive^67,72,73^. However, this effect is not observed genome-wide; it is largely restricted to regions containing CGIs^66,74^ and mediates the generation of large hypomethylated canyons or valleys across these CGI-rich domains, such as the *Hox* clusters^60,61^. The regulation of these hypomethylated, H3K27me3-marked domains involves a complex and still poorly understood interplay between PRC2, DNMT3A, and DNA demethylases (TET proteins) (Fig. 9a). There has been considerable focus on what defines the boundaries of these domains, which is thought to rely on a dynamic equilibrium between DNMT3A and TET proteins^60,61,70,71^. Knock-outs of either protein strongly affect domain boundaries, whilst the core regions remain protected through an unknown mechanism. The protection of these domains is exemplified by a *Dnmt3b* overexpression mouse model that shows aberrant gain of methylation genome-wide and a marginal increase (<10%) across H3K27me3-marked domains^75^. While DNMT3s have a strong affinity towards H3K36me2/3, there may be a weak affinity towards H3K27me3, such that the abundance of different DNMT3s and their interacting proteins could modulate chromatin localisation depending on the cellular context. A direct association of DNMTs with H3K27me3 regions in some contexts is supported by the observation that DNA methylation is dependent on the deposition of H3K27me3 at a subset of CGIs in extraembryonic ectoderm^76^ and cancer^77^. At this time, it is unclear how the D329A mutation may have altered the properties of DNMT3A to enhance its interaction with H3K27me3 and/or with members of the PRC2, allowing it not only to target the boundaries but the protected core of bivalent domains as well as (Fig. 9b). One possibility is that H3K36me2/3 acts as a sink for wild-type DNMT3A, and loss of affinity for this interaction would permit mutant protein to localise to bivalent chromatin exclusively, tipping the balance between DNMT3A and TETs at those sites.

As both DNA methylation and H3K27me3 are repressive modifications, it is rather surprising to see de-repression of strongly silenced genes in *Dnmt3a*^*Δ/D329A*^ hypothalamus, when methylation is acquired and H3K27me3 is maintained to some extent. In a similar observation, *Hox* genes were activated when DNA methylation was targeted to gene bodies within these regions in tumours^78^. Yet, in contrast, we find that DNA methylation is acquired across the whole domain, not just gene bodies. Together, these findings suggest that aberrant targeting of DNA methylation to the core of bivalent domains could lead to displacement of PRC2, disrupting the repressive chromatin conformation. This is supported by the observation that DNA methylation generally, albeit not always, inhibits PRC2 binding and activity in vitro^65,79^ and can block the deposition of H3K27me3^66^. Furthermore, aberrant methylation gain over PRC2 domains leads to loss of H3K27me3 when DNMT3B is overexpressed^75^. The H3K27me3 that remains at the hypermethylated DMRs we observed could be explained by lower histone turnover rates in non-replicative neurons or could be maintained by PRC2 variants that show affinity for methyl-CpG^79^.

Despite *Dnmt3a* being expressed throughout prenatal and postnatal development, we only observed hypermethylation of DNA in the presence of the *Dnmt3a*^*D329A*^ allele postnatally. In prenatal development, the predominant transcript isoform is *Dnmt3a2*, while in postnatal tissues it is *Dnmt3a1*^19,70^. The presence of the mutation specifically in the longer protein derived from isoform 1 may enable the aberrant targeting to bivalent chromatin, especially since DNMT3A1 has been found to associate with H3K27me3-marked shores of bivalent CGIs^70,71^. Alternatively, *Dnmt3b* is very highly expressed throughout early prenatal development and is responsible for the majority of de novo methylation^20^. Therefore, in this context, the aberrant localisation of the mutant DNMT3A may be rescued by heterodimerisation with DNMT3B^80,81^.

Recently, point mutations in the PWWP domain of human *DNMT3A* have been described in a specific class of tumour—hereditary paraganglioma^82^ and in microcephalic dwarfism^27^, including a substitution in the amino acid residue orthologous to murine D329. In both cases, the mutations are associated with gain of methylation of H3K27me3-marked domains containing developmental regulatory genes, similar to the findings in our study. Moreover, a W326R mutation induced in mice exhibits a similar dominant growth effect and hypermethylation, although the molecular mechanisms remain to be fully explored^27^. Importantly, in our study, the detailed analysis of the ectopic DNA methylation in relation to chromatin data suggests a positive recruitment of the DNMT3A-PWWP mutant protein to H3K27me3-marked domains, rather than non-selective relocalisation from H3K36me2/3 sites favoured by Heyn et al.^27^, as we do not observe gains of DNA methylation across other genome features. Nevertheless, future studies will be invaluable in elucidating the biochemical properties of the mutated DNMT3A PWWP domain.

In summary, we provide a characterisation of the growth phenotype and underlying molecular changes of the *Dnmt3a*^*D329A*^ mutant mouse model. We demonstrate that the mutant DNMT3A exhibits highly specific, altered targeting to H3K27me3-marked chromatin. The aberrant gain in DNA methylation across these domains is only observed in postnatal development, suggesting that the DNMT3A1 isoform may be uniquely affected or that the absence of DNMT3B in adult tissues unmasks this effect. This study provides further insights into the regulation of bivalent chromatin domains and the role of the DNMT3A PWWP domain. This model will be valuable for future investigations into the role of DNA methylation abnormalities in cancer and the growth regulatory function of DNMT3A.

## Methods

### Animal experimental procedures and sample collection

All animal experimental procedures were approved by the Animal Welfare and Ethical Review body at the Babraham Institute, and were conducted under authority of the UK Home Office issued licences in accordance with the Animal (Scientific Procedures) Act 1986. The *Dnmt3a*^*D329A*^ mutant strain was generated by GenOway (France) by targeting in C57BL/6 ES cells. Briefly, a point mutation GAT to GCT resulting in aspartic acid to alanine amino acid change at the endogenous locus of *Dnmt3a* codon 329 was introduced (Fig. 1a and Supplementary Fig. 1). Targeting documentation is available upon request. These mice were subsequently inter-crossed with C57BL/6Babr mice carrying a conditional *Dnmt3a* deletion allele *Dnmt3a*^*fl*^ and oocyte-specific *Zp3* promoter-driven *Cre* recombinase^12^. In order to obtain experimental litters *Dnmt3a*^*fl−/+*^ Zp3-*Cre*^*+ve*^ females, which carry oocytes with a conditionally deleted *Dnmt3a*^*∆/+*^ allele, were crossed with *Dnmt3a*^*D329A/+*^ males; yielding litters with four possible genotypes *Dnmt3a*^*+/+*^, *Dnmt3a*^*∆/+*^, *Dnmt3a*^*+/D329A*^, and *Dnmt3a*^*∆/D329A*^ (Fig. 1b). Matings, confirmed pregnancies, litters and pup numbers were recorded, where the *Dnmt3a*^*D329A*^ allele was passed through the maternal or paternal line in mating with wild-type C57BL/6Babr mice. Caesarean sections were performed to rescue the pups if *Dnmt3a*^*+/D329A*^ females failed to deliver. When possible, body weights of experimental litters were recorded at 1.4, 4, 6, 8, 10, and 12 weeks and analysed using mixed-model ANOVA with post hoc pairwise comparisons using Bonferroni correction. Litters were sacrificed and tissues were collected at embryonic day 7.5 (E7.5), postnatal day 1 (P1), P25, and adulthood (~14 weeks old). At E7.5 ectoplacental cone (EPC) and epiblast were collected in PBS and EZ nuclear lysis buffer (Sigma), respectively.

### Genotyping

E7.5 EPCs were lysed in embryo lysis buffer (1 M Tris pH8.5, 0.5 M EDTA pH 8, 10% Tween, 0.2 mg/ml Proteinase K) and used for genotyping by PCR with primers for *Dnmt3a*^*wt/fl/∆*^ alleles (D3A-F 5′-CTGTGGCATC TCAGGGTGAT GAGCA-3′, D3A-R1 5′-GCAAACAGAC CCAACATGGA ACCCT-3′, and D3A-R2 5′-TGAGTGGTGA GGCCCAGCTT ATCGA-3′) and *Dnmt3a*^*D329A*^ allele (D329A-F 5′-CAGATCCTTG CCTGAACTGT GGTGC-3′ and D329A-R 5′-TCCCTCTTGG TCCAGCATGT ACCCT-3′). Adult mice were genotyped by the same PCR assay using genomic DNA extracted from ear clips, or by service provider Transnetyx (US) using real-time PCR.

### ELISA

Blood samples from experimental animal hearts were collected into Microvette 500 Z-Gel tubes (Sartstedt), centrifuged for 5 min at 10,000 × *g* at RT. Serum collected was used in Rat/Mouse Growth Hormone ELISA kit (Millipore) and Quantikine ELISA for Mouse/Rat Igf-1 kit (R&D systems) as per manufacturers’ instructions.

### RNA-sequencing library preparation

Total RNA from 14-week-old animal hypothalami was extracted using TRIzol (Invitrogen) and cleaned using RNA Clean and Concentrator (Zymo research) and Ribo-Zero rRNA removal kit (Illumina). Libraries were generated using NEBNext Ultra RNA library preparation kits (New England Biolabs). RNA-seq libraries were sequenced 50 bp single-end using Illumina HiSeq2500 platform.

### RNA-seq analysis

RNA-seq data was trimmed using TrimGalore 0.5.0^83^, then mapped using HiSat2 2.1.0^84^ to *Mus musculus* genome GRCm38. Processed data was analysed using Seqmonk 1.42.0^85^. Reads over transcripts were merged across exons correcting for feature length, quantitated using RNA-Seq quantitation pipeline, and log_2_-transformed. Differentially expressed genes were called using DESeq with significance threshold of *p* < 0.01 after Benjamini–Hochberg multiple testing correction. Mitochondrial genes were excluded. For genome browser views of gene expression selected regions were split into 500 bp bins every 50 bp and quantitated as reads per million transcripts, unless specified otherwise. David 6.7^86^ Functional Annotation Tool was used for gene ontology analysis, with categories GOTERM_BP_FAT, GOTERM_CC_FAT, GOTERM_MF_FAT, using Benjamini-corrected *p* < 0.01 significance cut-off.

### PBAT sequencing library preparation

Post-bisulphite adaptor tagging (PBAT) was used to generate genome-wide DNA methylation profiles. Approximately 50 ng of gDNA or 10% of MNase-digested E7.5 Epiblast DNA, purified using solid phase reversible immobilisation (SPRI) beads, was used as an input material. Briefly, Imprint DNA modification kit (Sigma) one-step procedure was used to bisulphite-convert the DNA. First strand synthesis was done using Klenow exo-tagged (New England Biolabs) and biotin-tagged Illumina 9 bp random sequence adaptor, followed by Exo-I treatment (New England Biolabs) and SPRI purification. Biotinylated DNA was captured using Dynabeads Strepatavidin M-280 beads (ThermoFisher Scientific) and second strand synthesis was carried out using reverse Illumina 9 bp random sequence adaptor, followed by library amplification using Phusion HF (Thermo Scientific) or KAPA HiFi (Kapa Biosystems) polymerase for 10 cycles. Libraries were quantitated and quality control checked using Agilent High Sensitivity DNA kit and Kapa Illumina library quantitation kit, as per manufacturer’s instructions. Libraries were sequenced using 100 bp single-end for P1, P25, and adult tissues; and 150 bp for E7.5 epiblast sequencing mode on the Illumina NextSeq500 platform at the Babraham Institute Sequencing Facility.

### Methylation data analysis

Methylation data was trimmed using TrimGalore 0.5.0^83^, deduplicated, mapped to *Mus musculus* genome GRCm38, and methylation calls were extracted using Bismark 0.19.1^87^. Processed data was analysed using Seqmonk 1.42.0^85^. Pituitary datasets were down-sampled to the least covered dataset using SeqMonk to avoid differential coverage artefacts. Probes were defined as tiles of CpG positions and quantified using Bisulphite quantitation pipeline. Tiles in adult hypothalamus were 100-CpGs where at least 10 are covered. In pituitary, liver, E7.5 epiblast and when E7.5 epiblast data was used in analysis, tiles were 300-CpG where at least 10 are covered. Mitochondrial genes were excluded from analyses, X and Y genes were excluded only from E7.5 epiblast analyses. DMRs were called using Seqmonk edgeR (for/rev) statistics filter with absolute methylation difference cut-off of 20%, using multiple-testing corrected *p*-value of ≤ 0.01. Genomic locations were defined as listed in the brackets using feature annotations available in SeqMonk: promoter (−1500 to +500 bp around transcription start site), gene body (+500 bp to the end of the mRNA/gene), intergenic (promoters and gene bodies excluded). CGI features were called based on previously published CGI coordinate data^88^. For promoter analysis, 100CpG windows were fused together if distance between them were <1 kb. For intergenic regions, adjacent probes were fused together. Random probes were selected from all probes, excluding those in the DMR list, and utilised as representative of the genome. Previously described DNA methylation ‘canyon’ coordinates were used in the analysis^60^. Only those showing methylation under 10% and covered by at least 5 CpGs in hypothalamus were used in analysis. Non-CpG methylation was analysed using 1000CpH tiles where at least 30 CpHs were covered.

### Ultra-low input native ChIP-seq library preparation

Ultra-low input native chromatin immunoprecipitation sequencing (ChIP-seq) libraries were generated using 2.5% of a whole hypothalamus as input for each immunoprecipitation, following previously described protocol^89^, keeping 10% of that for input. Briefly, cells were permeabilised using triton X-100/deoxycholate, chromatin was then digested using micrococcal nuclease (New England Biolabs) and precleared using Dynabeads Protein A/G beads (ThermoFisher Scientific). Antibodies for H3K4me3 (250 ng per reaction, Diagenode C15410003), H3K27me3 (125 ng per reaction, Millipore 07-449), or H3K36me3 (250 ng per reaction, Diagenode C15410192) were pre-bound to Protein A/G beads and incubated with chromatin for immunoprecipitation. Bound DNA was purified by SPRI purification. MicroPlex Library Preparation kit v2 (Diagenode) was used as per manufacturer’s instructions to generate libraries of inputs and immunoprecipitated samples. Libraries were quantitated and quality control checked using Agilent High Sensitivity DNA kit and Kapa Illumina library quantitation kit, as per manufacturer’s instructions. Libraries were sequenced using 75 bp single-end sequencing on the Illumina NextSeq500 platform at the Babraham Institute Sequencing Facility.

### ChIP data analysis

ChIP-seq data was trimmed using Trim Galore 0.5.0^83^ and mapped against *Mus Musculus* GRCm38 genome with Bowtie 1.2.2^90^. H3K27me3 enrichment was quantitated using 2 kb tiles with a 1 kb step, correcting for total count based on the largest library, unless stated otherwise. H3K4me3 and H3K27me3 peaks were called using MACS peak caller within SeqMonk 1.42.0, using a significant threshold of *p* < 0.00001, a sonication fragment size of 1 kb, and input controls as a reference. When assessment of histone marks was done in relation to DNA methylation, DNA methylation tiles were used to quantitate the enrichment.

### Statistical analyses

All measurements taken were biological and not technical replicates, replicates represent individual animals. Basic statistical tests used are described in figure legends where applicable.

### Reporting summary

Further information on experimental design is available in the Nature Research Reporting Summary linked to this article.

## Supplementary information

Supplementary Information

Peer Review File

Reporting Summary

Description of Additional Supplementary Files

Supplementary Data 1

Supplementary Data 2

Supplementary Data 3

Supplementary Data 4

Supplementary Data 5

Supplementary Data 6

Supplementary Data 7

Supplementary Data 8

Supplementary Data 9

Supplementary Data 10

Supplementary Data 11

Supplementary Data 12

Supplementary Data 13

Supplementary Data 14

Source Data

## Data Availability

Sequence data that support the findings of this study have been deposited in Gene Expression Omnibus with the primary accession code GSE117728. All data generated and analysed in this study are included in the published article and its supplementary files and are available from the corresponding author upon reasonable request. Source data for Figs. 1c, 2a, 3a, 4a, b, 6e–i, 8b, c and Supplementary Figs. 3, 4a, 4c, d, 5a, b, 11a, 11f–i are provided. Source data for Figs. 2c, d and 5b are provided in Supplementary Data. A Life Science and ChIP-Seq Reporting Summary for this article is available.

## References

[1] Smith ZD, Meissner A (2013). DNA methylation: roles in mammalian development. Nat. Rev. Genet..

[2] Yin Y (2017). Impact of cytosine methylation on DNA binding specificities of human transcription factors. Science.

[3] Borgel J (2010). Targets and dynamics of promoter DNA methylation during early mouse development. Nat. Genet..

[4] Jones PA (2012). Functions of DNA methylation: islands, start sites, gene bodies and beyond. Nat. Rev. Genet..

[5] Smith ZD (2012). A unique regulatory phase of DNA methylation in the early mammalian embryo. Nature.

[6] Ziller MJ (2013). Charting a dynamic DNA methylation landscape of the human genome. Nature.

[7] Ehrlich M, Wang RY (1981). 5-Methylcytosine in eukaryotic DNA. Science.

[8] Okano M, Xie S, Li E (1998). Cloning and characterization of a family of novel mammalian DNA (cytosine-5) methyltransferases. Nat. Genet..

[9] Barau J (2016). The DNA methyltransferase DNMT3C protects male germ cells from transposon activity. Science.

[10] Jain D (2017). rahu is a mutant allele of Dnmt3c, encoding a DNA methyltransferase homolog required for meiosis and transposon repression in the mouse male germline. PLoS. Genet..

[11] Li E, Bestor TH, Jaenisch R (1992). Targeted mutation of the DNA methyltransferase gene results in embryonic lethality. Cell.

[12] Chuang LSH (1997). Human DNA-(cytosine-5) methyltransferase–PCNA complex as a target for p21WAF1. Science.

[13] Bostick M (2007). UHRF1 plays a role in maintaining DNA methylation in mammalian cells. Science.

[14] Sharif J (2007). The SRA protein Np95 mediates epigenetic inheritance by recruiting Dnmt1 to methylated DNA. Nature.

[15] Bourc’his D, Xu GL, Lin CS, Bollman B, Bestor TH (2001). Dnmt3L and the establishment of maternal genomic imprints. Science.

[16] Hata K, Okano M, Lei H, Li E (2002). Dnmt3L cooperates with the Dnmt3 family of de novo DNA methyltransferases to establish maternal imprints in mice. Development.

[17] Bourc’his D, Bestor TH (2004). Meiotic catastrophe and retrotransposon reactivation in male germ cells lacking Dnmt3L. Nature.

[18] Okano M, Bell DW, Haber DA, Li E (1999). DNA methyltransferases Dnmt3a and Dnmt3b are essential for de novo methylation and mammalian development. Cell.

[19] Chen T, Ueda Y, Xie S, Li E (2002). A novel Dnmt3a isoform produced from an alternative promoter localizes to euchromatin and its expression correlates with activede novo methylation. J. Biol. Chem..

[20] Auclair G, Guibert S, Bender A, Weber M (2014). Ontogeny of CpG island methylation and specificity of DNMT3 methyltransferases during embryonic development in the mouse. Genome Biol..

[21] Kaneda M (2004). Essential role for de novo DNA methyltransferase Dnmt3a in paternal and maternal imprinting. Nature.

[22] Kaneda M (2010). Genetic evidence for Dnmt3a-dependent imprinting during oocyte growth obtained by conditional knockout with Zp3-Cre and complete exclusion of Dnmt3b by chimera formation. Genes Cells.

[23] Feng J, Chang H, Li E, Fan G (2005). Dynamic expression of de novo DNA methyltransferases Dnmt3a and Dnmt3b in the central nervous system. J. Neurosci. Res..

[24] Tatton-Brown K (2014). Mutations in the DNA methyltransferase gene DNMT3A cause an overgrowth syndrome with intellectual disability. Nat. Genet..

[25] Yang L, Rau R, Goodell MA (2015). DNMT3A in haematological malignancies. Nat. Rev. Cancer.

[26] Tatton-Brown K (2017). Mutations in epigenetic regulation genes are a major cause of overgrowth with intellectual disability. Am. J. Hum. Genet..

[27] Heyn P (2019). Gain-of-function DNMT3A mutations cause microcephalic dwarfism and hypermethylation of Polycomb-regulated regions. Nat. Genet..

[28] Hansen RS (1999). The DNMT3B DNA methyltransferase gene is mutated in the ICF immunodeficiency syndrome. Proc. Natl Acad. Sci. USA.

[29] Dodge JE, Ramsahoye BH, Wo ZG, Okano M, Li E (2002). De novo methylation of MMLV provirus in embryonic stem cells: CpG versus non-CpG methylation. Gene.

[30] Ramsahoye BH (2000). Non-CpG methylation is prevalent in embryonic stem cells and may be mediated by DNA methyltransferase 3a. Proc. Natl Acad. Sci. USA.

[31] Wienholz BL (2010). DNMT3L modulates significant and distinct flanking sequence preference for DNA methylation by DNMT3A and DNMT3B in vivo. PLoS Genet..

[32] Jeltsch A, Broche J, Bashtrykov P (2018). Molecular processes connecting DNA methylation patterns with DNA methyltransferases and histone modifications in mammalian genomes. Genes.

[33] Argentaro A (2007). Structural consequences of disease-causing mutations in the ATRX-DNMT3-DNMT3L (ADD) domain of the chromatin-associated protein ATRX. PNAS.

[34] Otani J (2009). Structural basis for recognition of H3K4 methylation status by the DNA methyltransferase 3A ATRX–DNMT3–DNMT3L domain. EMBO Rep..

[35] Ooi SKT (2007). DNMT3L connects unmethylated lysine 4 of histone H3 to de novo methylation of DNA. Nature.

[36] Zhang Y (2010). Chromatin methylation activity of Dnmt3a and Dnmt3a/3L is guided by interaction of the ADD domain with the histone H3 tail. Nucleic Acids Res..

[37] Guo X (2015). Structural insight into autoinhibition and histone H3-induced activation of DNMT3A. Nature.

[38] Li BZ (2011). Histone tails regulate DNA methylation by allosterically activating de novo methyltransferase. Cell Res..

[39] Qin S, Min J (2014). Structure and function of the nucleosome-binding PWWP domain. Trends Biochem. Sci..

[40] Chen T, Tsujimoto N, Li E (2004). The PWWP domain of Dnmt3a and Dnmt3b Is required for directing DNA methylation to the major satellite repeats at pericentric heterochromatin. Mol. Cell. Biol..

[41] Ge YZ (2004). Chromatin targeting of de Novo DNA methyltransferases by the PWWP domain. J. Biol. Chem..

[42] Shirohzu H (2002). Three novel DNMT3B mutations in Japanese patients with ICF syndrome. Am. J. Med. Genet..

[43] Rondelet G, Dal Maso T, Willems L, Wouters J (2016). Structural basis for recognition of histone H3K36me3 nucleosome by human de novo DNA methyltransferases 3A and 3B. J. Struct. Biol..

[44] Dhayalan A (2010). The Dnmt3a PWWP domain reads histone 3 lysine 36 trimethylation and guides DNA methylation. J. Biol. Chem..

[45] Bock I (2011). Application of Celluspots peptide arrays for the analysis of the binding specificity of epigenetic reading domains to modified histone tails. BMC Biochem..

[46] Kungulovski G (2014). Application of histone modification-specific interaction domains as an alternative to antibodies. Genome Res..

[47] Mauser R, Kungulovski G, Keup C, Reinhardt R, Jeltsch A (2017). Application of dual reading domains as novel reagents in chromatin biology reveals a new H3K9me3 and H3K36me2/3 bivalent chromatin state. Epigenetics Chromatin.

[48] Baubec T (2015). Genomic profiling of DNA methyltransferases reveals a role for DNMT3B in genic methylation. Nature.

[49] Morselli M (2015). In vivo targeting of de novo DNA methylation by histone modifications in yeast and mouse. eLife.

[50] Greenberg MVC (2017). Transient transcription in the early embryo sets an epigenetic state that programs postnatal growth. Nat. Genet..

[51] Li G (2014). Major epigenetic development distinguishing neuronal and non-neuronal cells occurs postnatally in the murine hypothalamus. Hum. Mol. Genet..

[52] Abizaid A, Horvath TL (2008). Brain circuits regulating energy homeostasis. Regul. Pept..

[53] Leibel R (2008). Molecular physiology of weight regulation in mice and humans. Int. J. Obes..

[54] Mihalache L (2016). Effects of ghrelin in energy balance and body weight homeostasis. Hormones.

[55] Yang H, An JJ, Sun C, Xu B (2016). Regulation of energy balance via BDNF expressed in nonparaventricular hypothalamic neurons. Mol. Endocrinol..

[56] Li X (2013). SIRT1 and energy metabolism. Acta Biochim. Biophys. Sin..

[57] Lau, J. & Herzog, H. CART in the regulation of appetite and energy homeostasis. *Front. Neurosci*. **8**, 313 (2014). 10.3389/fnins.2014.00313 PMC4195273 25352770

[58] Plant TM (2015). 60 YEARS OF NEUROENDOCRINOLOGY: the hypothalamo-pituitary–gonadal axis. J. Endocrinol..

[59] Borgel J (2010). Targets and dynamics of promoter DNA methylation during early mouse development. Nat. Genet..

[60] Jeong M (2014). Large conserved domains of low DNA methylation maintained by Dnmt3a. Nat. Genet..

[61] Li Y (2018). Genome-wide analyses reveal a role of Polycomb in promoting hypomethylation of DNA methylation valleys. Genome Biol..

[62] Boyer LA (2006). Polycomb complexes repress developmental regulators in murine embryonic stem cells. Nature.

[63] Tanay A, O’Donnell AH, Damelin M, Bestor TH (2007). Hyperconserved CpG domains underlie Polycomb-binding sites. PNAS.

[64] Lindroth AM (2008). Antagonism between DNA and H3K27 Methylation at the Imprinted Rasgrf1 Locus. PLoS Genet..

[65] Bartke T (2010). Nucleosome-interacting proteins regulated by DNA and histone methylation. Cell.

[66] Brinkman AB (2012). Sequential ChIP-bisulfite sequencing enables direct genome-scale investigation of chromatin and DNA methylation cross-talk. Genome Res..

[67] Xie W (2012). Base-resolution analyses of sequence and parent-of-origin dependent DNA methylation in the mouse genome. Cell.

[68] Varley KE (2013). Dynamic DNA methylation across diverse human cell lines and tissues. Genome Res..

[69] Lister R (2013). Global epigenomic reconfiguration during mammalian brain development. Science.

[70] Manzo M (2017). Isoform-specific localization of DNMT3A regulates DNA methylation fidelity at bivalent CpG islands. EMBO J..

[71] Gu T (2018). DNMT3A and TET1 cooperate to regulate promoter epigenetic landscapes in mouse embryonic stem cells. Genome Biol..

[72] Bogdanović O (2011). Temporal uncoupling of the DNA methylome and transcriptional repression during embryogenesis. Genome Res..

[73] Murphy PJ (2013). Single-molecule analysis of combinatorial epigenomic states in normal and tumor cells. PNAS.

[74] Statham AL (2012). Bisulfite sequencing of chromatin immunoprecipitated DNA (BisChIP-seq) directly informs methylation status of histone-modified DNA. Genome Res..

[75] Zhang Y (2018). Targets and genomic constraints of ectopic Dnmt3b expression. eLife.

[76] Smith ZD (2017). Epigenetic restriction of extraembryonic lineages mirrors the somatic transition to cancer. Nature.

[77] Schlesinger Y (2007). Polycomb-mediated methylation on Lys27 of histone H3 pre-marks genes for de novo methylation in cancer. Nat. Genet..

[78] Su J (2018). Homeobox oncogene activation by pan-cancer DNA hypermethylation. Genome. Biol..

[79] Wang X (2017). Molecular analysis of PRC2 recruitment to DNA in chromatin and its inhibition by RNA. Nat. Struct. Mol. Biol..

[80] Li JY (2007). Synergistic function of DNA methyltransferases Dnmt3a and Dnmt3b in the methylation of Oct4 and Nanog. Mol. Cell. Biol..

[81] Emperle M, Rajavelu A, Reinhardt R, Jurkowska RZ, Jeltsch A (2014). Cooperative DNA binding and protein/DNA fiber formation increases the activity of the Dnmt3a DNA methyltransferase. J. Biol. Chem..

[82] Remacha L (2018). Gain-of-function mutations in DNMT3A in patients with paraganglioma. Genet. Med..

[83] Babraham Bioinformatics—Trim Galore! http://www.bioinformatics.babraham.ac.uk/projects/trim_galore/. Accessed 10 Mar 2019.

[84] Kim D, Langmead B, Salzberg SL (2015). HISAT: a fast spliced aligner with low memory requirements. Nat Meth.

[85] Babraham Bioinformatics*. SeqMonk Mapped Sequence Analysis Tool*. http://www.bioinformatics.babraham.ac.uk/projects/seqmonk/. Accessed 20 June 2017.

[86] DAVID Functional Annotation Bioinformatics Microarray Analysis. https://david-d.ncifcrf.gov/. Accessed 10 Mar 2019.

[87] Krueger F, Andrews SR (2011). Bismark: a flexible aligner and methylation caller for Bisulfite-Seq applications. Bioinformatics.

[88] Illingworth Robert S., Gruenewald-Schneider Ulrike, Webb Shaun, Kerr Alastair R. W., James Keith D., Turner Daniel J., Smith Colin, Harrison David J., Andrews Robert, Bird Adrian P. (2010). Orphan CpG Islands Identify Numerous Conserved Promoters in the Mammalian Genome. PLoS Genetics.

[89] Hanna CW (2018). MLL2 conveys transcription-independent H3K4 trimethylation in oocytes. Nat. Struct. Mol. Biol..

[90] Langmead B, Trapnell C, Pop M, Salzberg SL (2009). Ultrafast and memory-efficient alignment of short DNA sequences to the human genome. Genome Biol..

