# Supplementary material for: A DNMT3A PWWP mutation leads to methylation of bivalent chromatin and growth retardation in mice
Source: Nat Commun. 2019 Apr 23;10:1884. doi: 10.1038/s41467-019-09713-w (PMC6478690; doi:10.1038/s41467-019-09713-w)
Supplement: Supplementary file 1 — Supplementary Information [file 41467_2019_9713_MOESM1_ESM.pdf]

Supplementary Information

A DNMT3A PWWP mutation leads to methylation of bivalent chromatin and growth retardation in mice

Sendžikaitė et al.

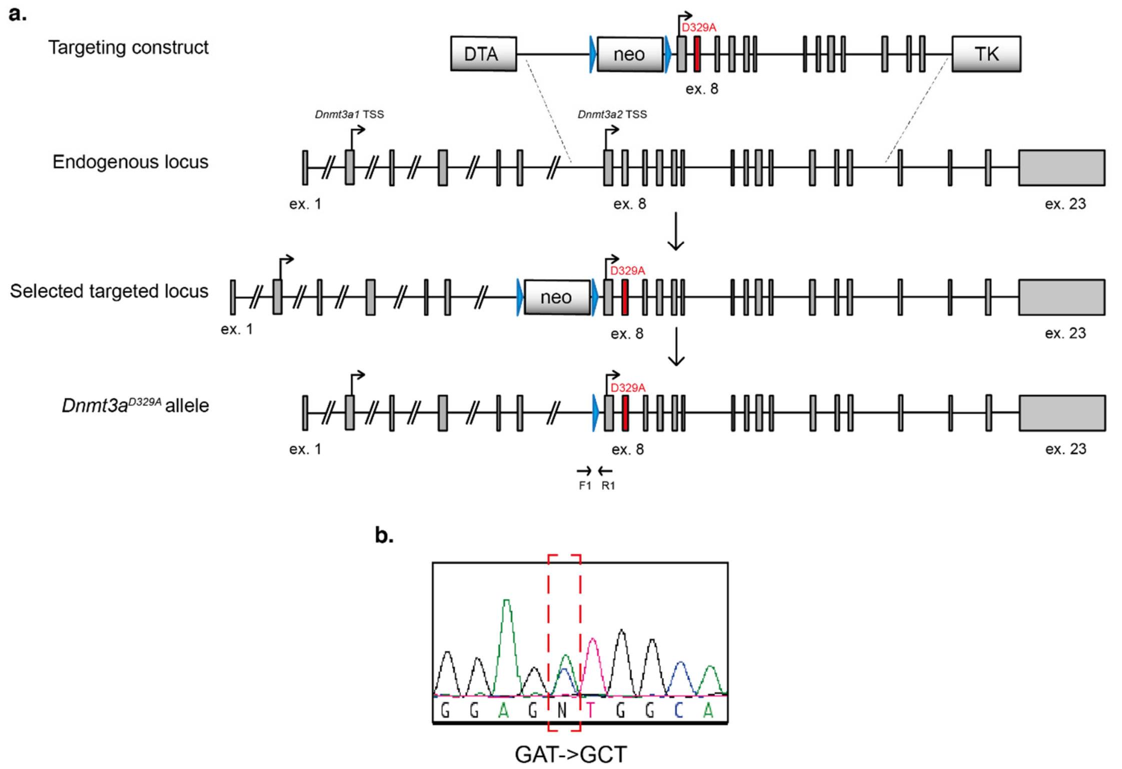

Supplementary Figure 1. Generation of the *Dnmt3a*^{D329A} allele.

a, Schematic representation of targeting construct used to generate mice carrying the *Dnmt3a*^{D329A} mutation. Targeting construct, endogenous locus and selected targeted locus are shown together with final neo cassette-excised mutant allele. Exon 8 with the D329A mutation is coloured in red; *loxP* sites indicated as blue arrowheads. Abbreviations: DTA: diptheria toxin A negative selection cassette, neo: neomycin selection cassette, TK: herpes simplex virus thymidine kinase, TSS: transcription start site, ex.: exon. F1 and R1 indicate primers used to genotype the mutant allele.

b, Sanger sequencing screenshot from genomic DNA of a *Dnmt3a*$^{+/D329A}$ mouse. Boxed region highlights the heterozygous base position.

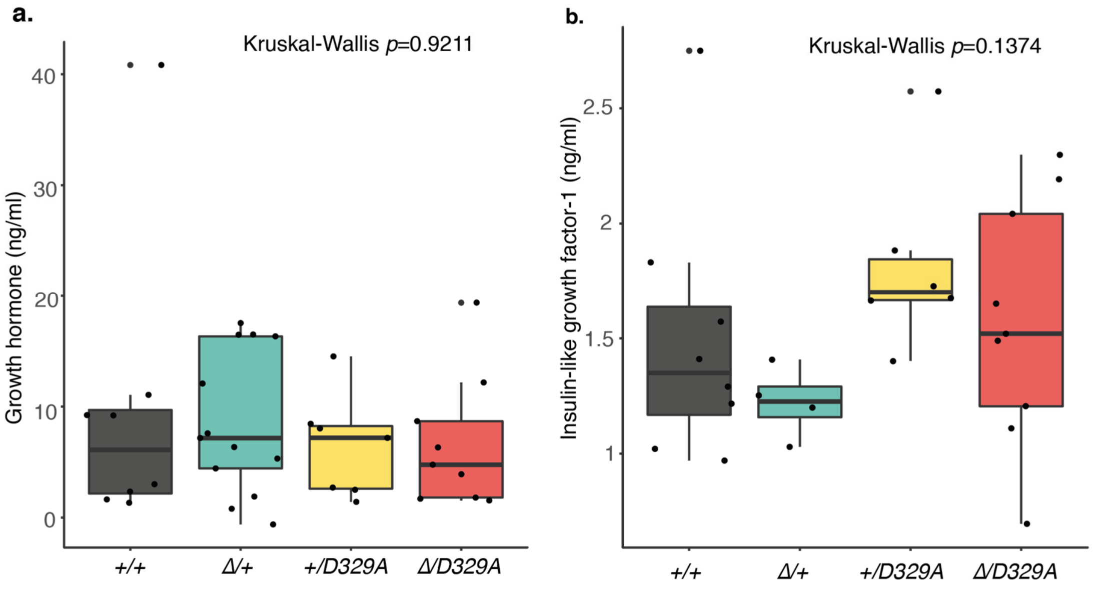

Supplementary Figure 2. Serum hormone levels in *$Dnmt3a^{D329A}$* mice.
a, Levels of growth hormone determined by ELISA in serum of 14-week old male mice of the indicated *Dnmt3a* genotypes. *n*(+/+)=8, *n*(*Δ*/+) = 13, *n*(+/*D329A*) = 7, *n*(*Δ*/*D329A*) = 9. Kruskal-Wallis non-parametric test (*p*=0.9211, H=0.49, df=3).
b, Levels of insulin-like growth factor-1 determined by ELISA in serum of 14-week old animals of indicated *Dnmt3a* genotype. *n*(+/+) = 8, *n*(*Δ*/+) = 4, *n*(+/*D329A*) = 6, *n*(*Δ*/*D329A*) = 9. Kruskal-Wallis non-parametric test (*p*=0.1374, H=5.52, df = 3).
In **a**,**b**, Boxplots show median value and 25-75th percentiles, whiskers show lowest and highest observation, excluding outliers. Points represent individual animals.

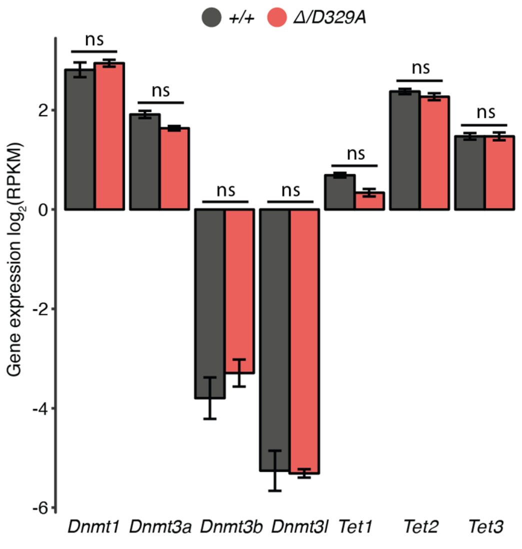

Supplementary Figure 3. Expression of *Dnmt* and *Tet* family genes.
Abundance of transcripts for *Dnmt* and *Tet* genes in RNA-seq data from female adult (14-week) hypothalamus. *n*(+/+) = 5, *n*(*Δ*/*D392A*) = 4. Two-tailed t-test was used with Bonferroni-corrected *p*-value<0.5 significance cut-off. ns.: not significant. Error bars indicate standard deviation. Raw data are provided in Source Data.

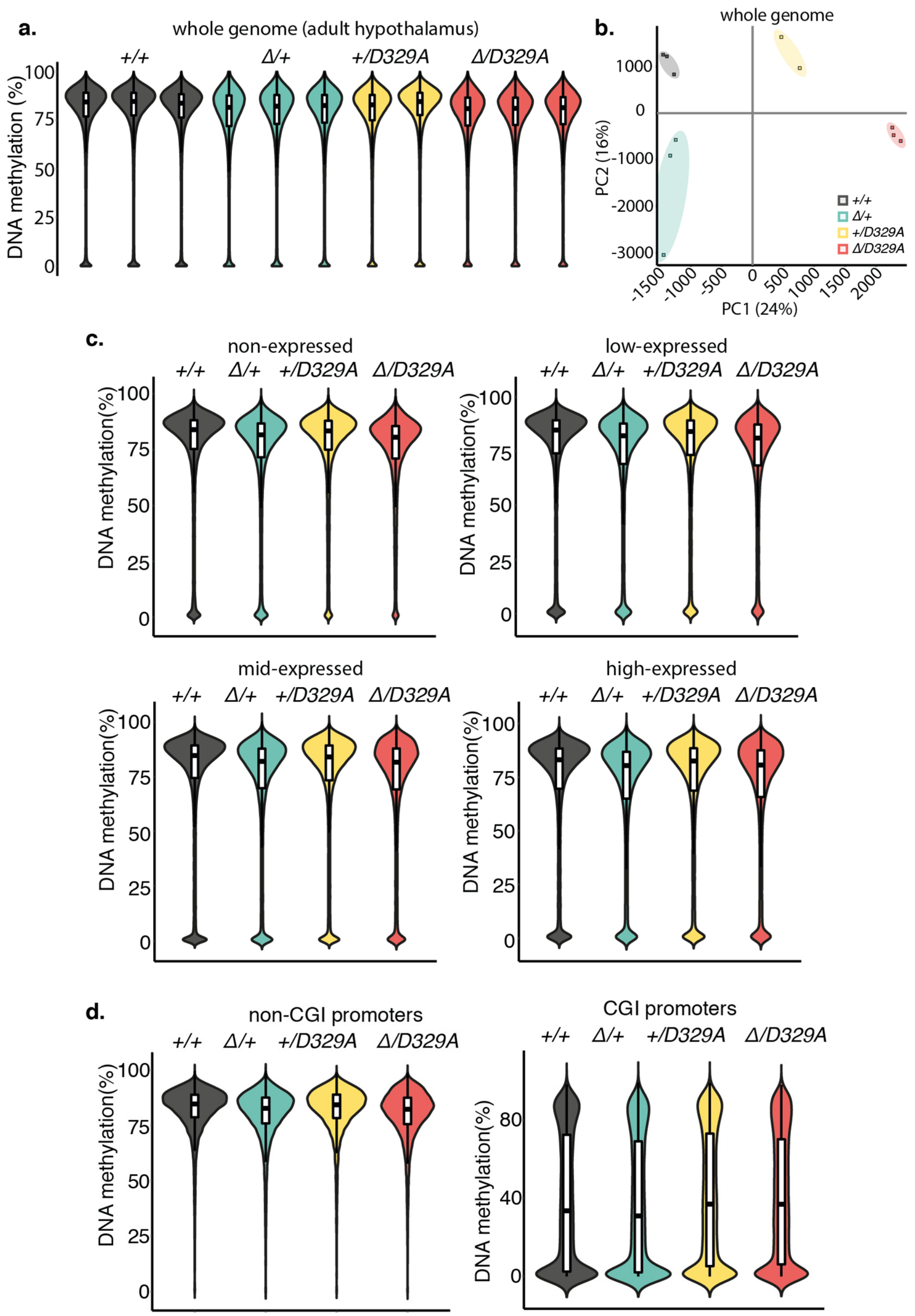

Supplementary Figure 4. Global DNA methylation trends in mice carrying *Dnmt3a*^{D329A} allele.

a, Beanplots indicating whole genome methylation levels in adult (14-week) male hypothalamus of mice carrying the alleles shown. Each plot represents the data from an individual mouse. Quantification of methylation was for tiles of 100-CpG positions.

b, PCA plot showing clustering of individual samples into separate distant groups based on the genotype. PC1 separation appears to be driven by mutant allele, whilst PC2 separation is driven by number of alleles expressed.
c, Beanplots indicating methylation levels quantified over genes, split by their expression levels. Non-expressed: $\log_2$(RPKM) < 0; low-expressed: 0 < $\log_2$(RPKM) <1; mid-expressed: 1 < $\log_2$(RPKM) <3; high: expressed $\log_2$(RPKM) >3.
d, Beanplots indicating methylation levels quantified over CGI non-overlapping or overlapping promoters. Probes within 1kb distance were merged. CGI: CpG island.
In **a-d**, *n*(*+/+, Δ/+, Δ/D329A*) = 3, *n*(*+/D329A*) = 2; in **a**,**c**,**d**, Boxplots show median value and 25-75th percentiles, whiskers show lowest and highest observation, excluding outliers. Raw data are provided in Source Data.

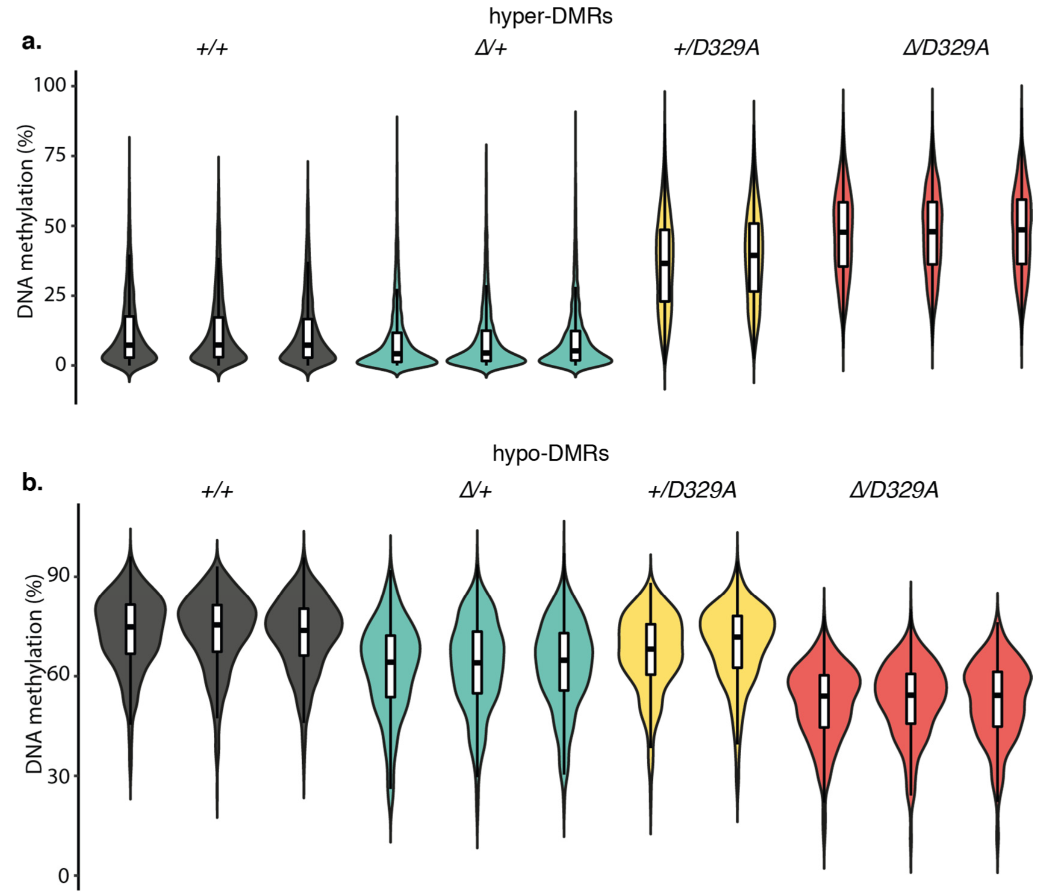

Supplementary Figure 5. Differential methylation in *Dnmt3a*$^{+/+}$, *Dnmt3a*$^{Δ/+}$, *Dnmt3a*$^{+/D329A}$ and *Dnmt3a*$^{Δ/D329A}$ hypothalamus.
a,**b**, Beanplots showing reproducible DNA methylation changes over (**a**) hyper- and (**b**) hypo- DMRs in 14-week adult male hypothalamus across four different genotypes. Boxplots show median value and 25-75th percentiles, whiskers show lowest and highest observation, excluding outliers. Each plot represents the data from an individual mouse. Hypo: hypomethylated, hyper: hypermethylated, DMR: differentially methylated region.
In **a,b**, *n*(*+/+, Δ/+, Δ/D329A*) = 3, *n*(*+/D329A*) = 2. Raw data are provided in Source Data.

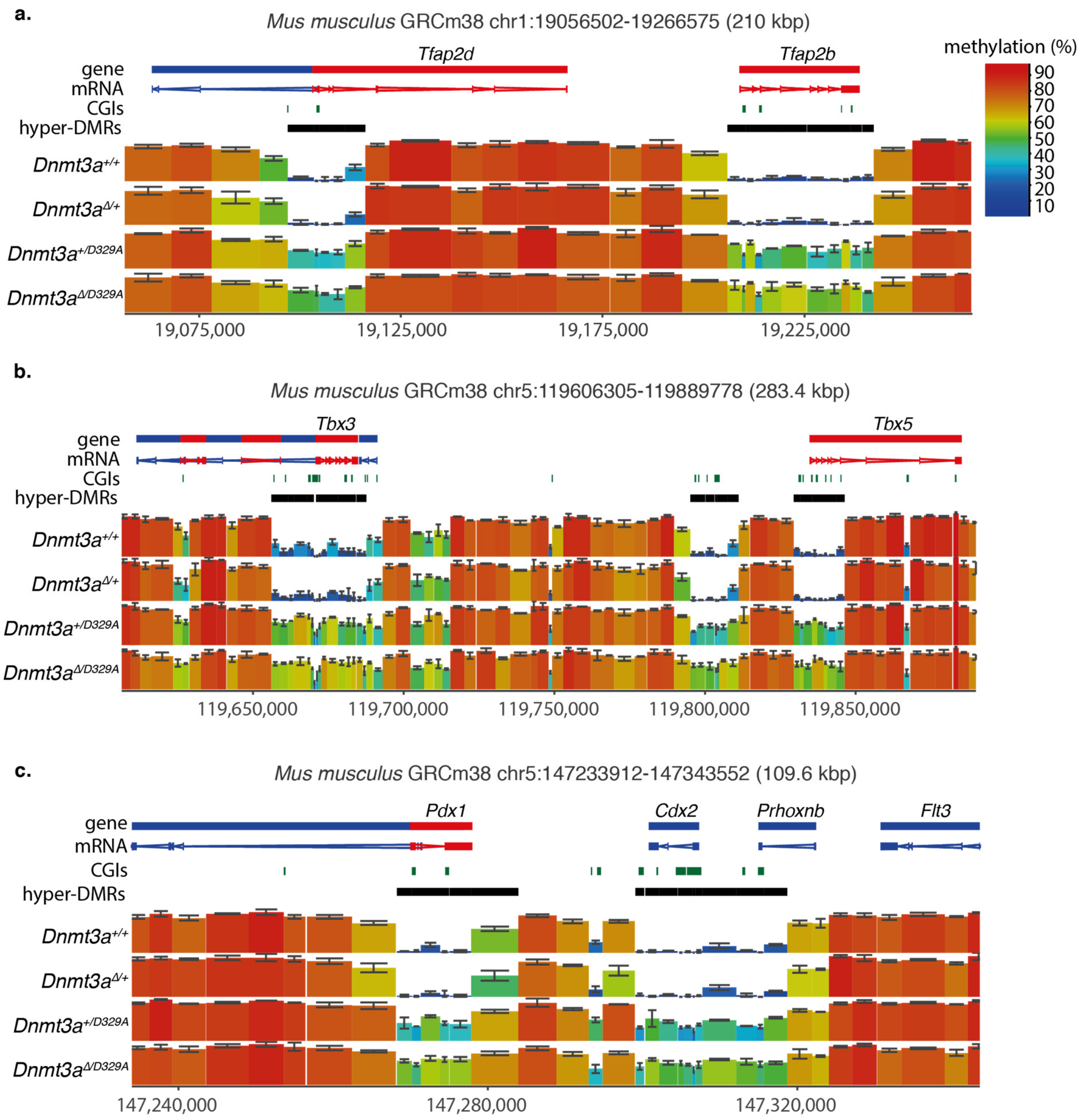

Supplementary Figure 6. Genome browser views of representative regions that gain methylation in *Dnmt3a*$^{\Delta/D392A}$ hypothalamus. a, **b**, and **c**, show three representative affected loci. Methylation profiles for 14-week adult male hypothalamus for each *Dnmt3a* genotype are shown. Each block, colour-coded for methylation value, represents a 100-CpG tile. Abbreviations: CGIs: CpG islands, Hyper-DMRs: hypermethylated regions. Gain of methylation is evident in both *Dnmt3a*$^{+/D392A}$ and *Dnmt3a*$^{\Delta/D392A}$. For gene and mRNA tracks, the colour indicates direction, where red is a forward strand and blue is a reverse strand. $n(+/+, \Delta/+, \Delta/D329A) = 3$, $n(+/D329A) = 2$. Error bars indicate standard deviation.

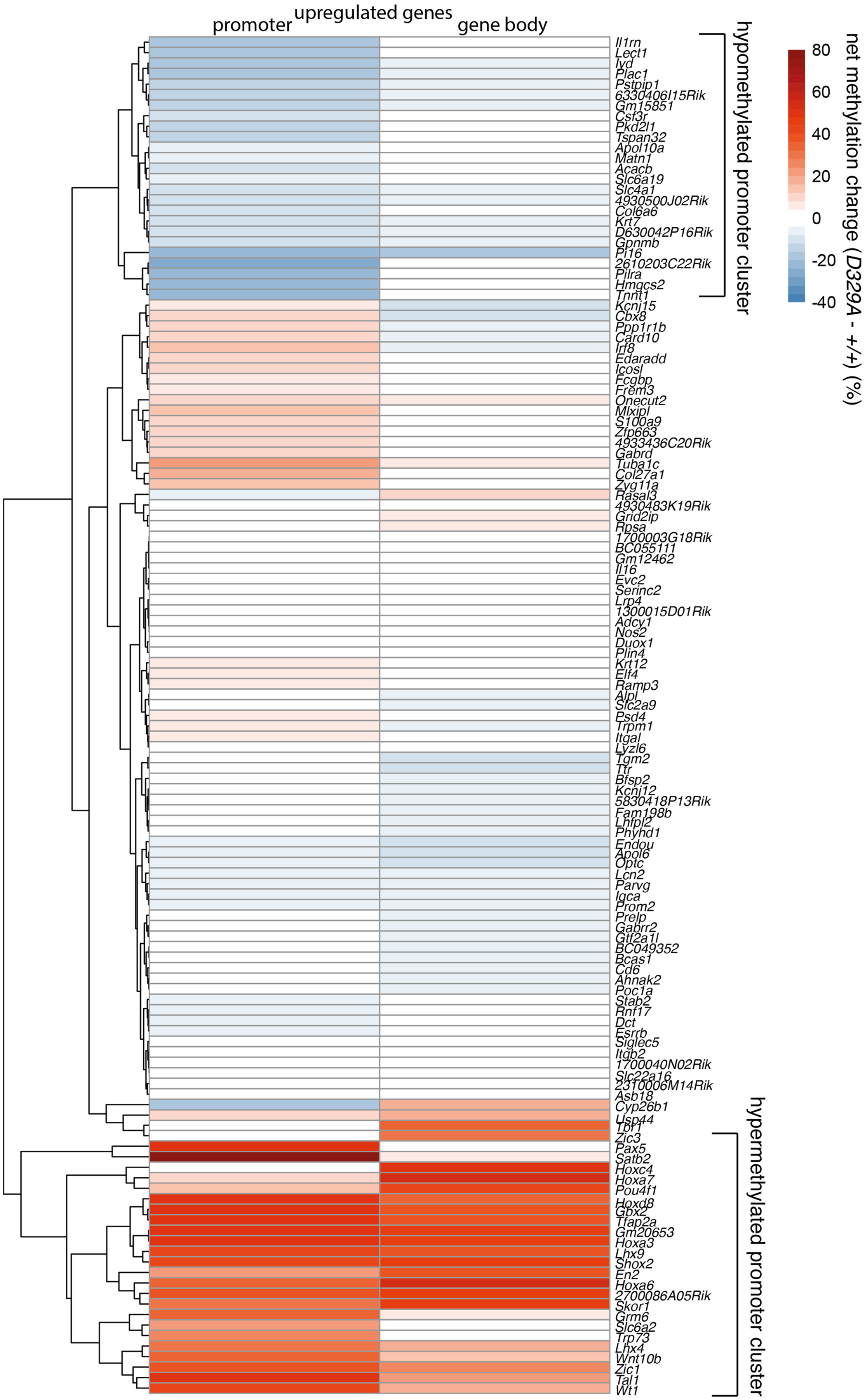
upregulated genes
promoter
gene body
hypomethylated promoter cluster
hypermethylated promoter cluster
net methylation change (D329A - +/+) (%)
80
60
40
20
0
-20
-40
Il1rn
Lect1
Ivd
Plac1
Pstpip1
6330406I15Rik
Gm15851
Csf3r
Pkd2l1
Tspan32
Apol10a
Matn1
Acacb
Slc6a19
Slc4a1
4930500J02Rik
Col6a6
Krt7
D630042P16Rik
Gpnmb
Pi16
2610203C22Rik
Pilra
Hmgcs2
Tnnt1
Kcnj15
Cbx8
Ppp1r1b
Card10
Irf8
Edaradd
Icosl
Fcgbp
Frem3
Onecut2
Mlxipl
S100a9
Zfp663
4933436C20Rik
Gabrd
Tuba1c
Col27a1
Zyg11a
Rasal3
4930483K19Rik
Grid2ip
Rpsa
1700003G18Rik
BC055111
Gm12462
Il16
Evc2
Serinc2
Lrp4
1300015D01Rik
Adcy1
Nos2
Duox1
Plin4
Krt12
Elf4
Ramp3
Alpl
Slc2a9
Psd4
Trpm1
Itgal
Lyzl6
Tgm2
Ttr
Bfsp2
Kcnj12
5830418P13Rik
Fam198b
Lhfpl2
Phyhd1
Endou
Apol6
Optc
Lcn2
Parvg
Igca
Prom2
Prelp
Gabrr2
Gtf2a1l
BC049352
Bcas1
Cd6
Ahnak2
Poc1a
Stab2
Rnf17
Dct
Esrrb
Siglec5
Itgb2
1700040N02Rik
Slc22a16
2310006M14Rik
Asb18
Cyp26b1
Usp44
Tbr1
Zic3
Pax5
Satb2
Hoxc4
Hoxa7
Pou4f1
Hoxd8
Gbx2
Tfap2a
Gm20653
Hoxa3
Lhx9
Shox2
En2
Hoxa6
2700086A05Rik
Skor1
Grm6
Slc6a2
Trp73
Lhx4
Wnt10b
Zic1
Tal1
Wt1

Supplementary Figure 7. Net methylation changes over promoters and gene bodies for genes upregulated in *Dnmt3a*$^{\Delta/D329A}$ adult hypothalamus. Methylation differences between *Dnmt3a*$^{+/+}$ and *Dnmt3a*$^{\Delta/D392A}$ were quantified over promoters and gene bodies. Promoter probes within 1kb distance were merged. Clustering was performed using the Euclidean complete method. $n(+/+, \Delta/D329A) = 3$.

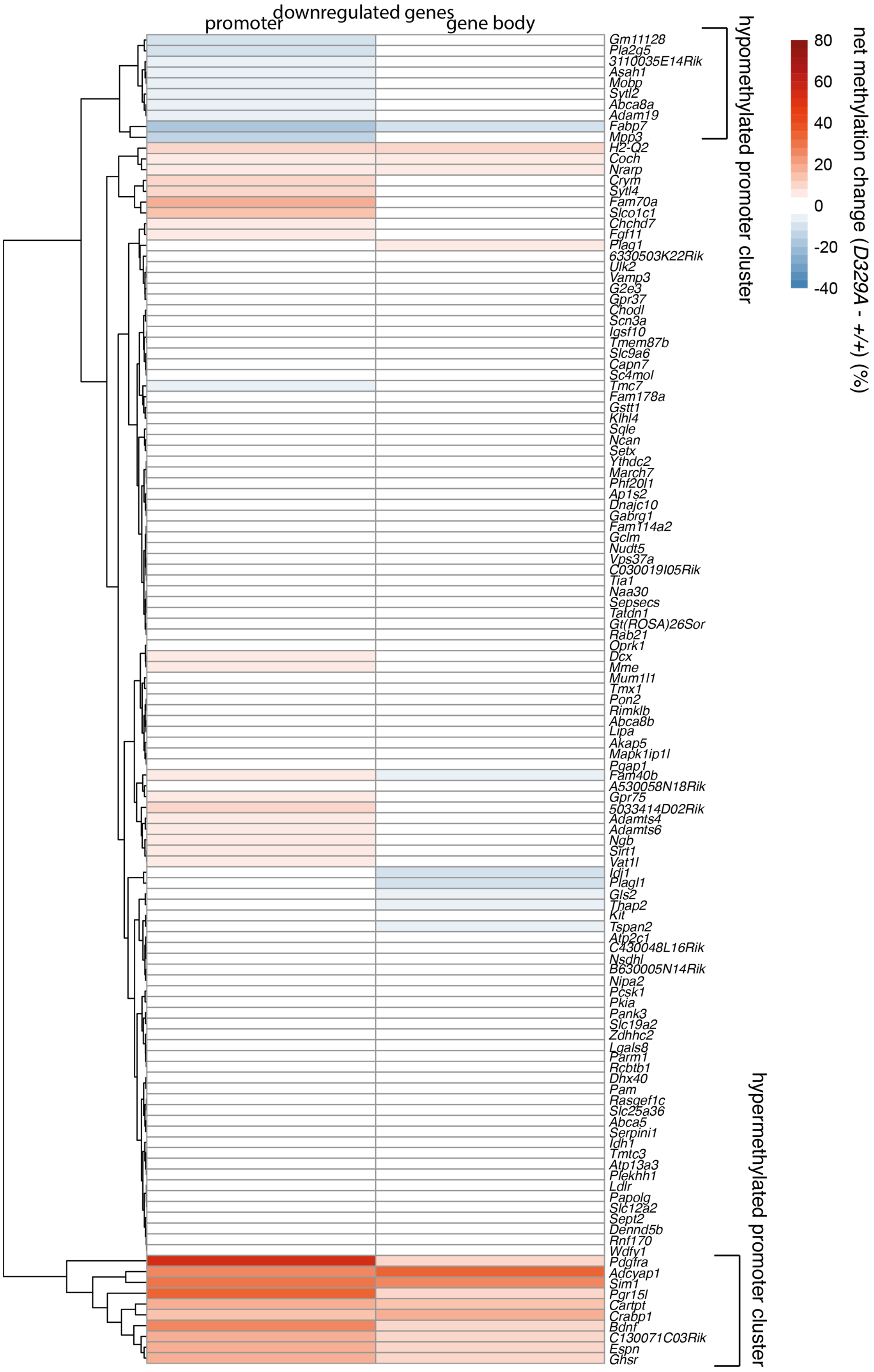
downregulated genes
promoter
gene body
hypomethylated promoter cluster
hypermethylated promoter cluster
net methylation change (D329A - +/+) (%)
80
60
40
20
0
-20
-40
Gm11128
Pla2g5
3110035E14Rik
Asah1
Mobp
Sytl2
Abca8a
Adam19
Fabp7
Mpp3
H2-Q2
Coch
Nrarp
Crym
Sytl4
Fam70a
Slco1c1
Chchd7
Fgf11
Plag1
6330503K22Rik
Ulk2
Vamp3
G2e3
Gpr37
Chodl
Scn3a
Igsf10
Tmem87b
Slc9a6
Capn7
Sc4mol
Tmc7
Fam178a
Gstt1
Klhl4
Sqle
Ncan
Setx
Ythdc2
March7
Phf20l1
Ap1s2
Dnajc10
Gabrg1
Fam114a2
Gclm
Nudt5
Vps37a
C030019I05Rik
Tia1
Naa30
Sepsecs
Tatdn1
Gt(ROSA)26Sor
Rab21
Oprk1
Dcx
Mme
Mum1l1
Tmx1
Pon2
Rimklb
Abca8b
Lipa
Akap5
Mapk1ip1l
Pgap1
Fam40b
A530058N18Rik
Gpr75
5033414D02Rik
Adamts4
Adamts6
Ngb
Sirt1
Vat1l
Idi1
Plagl1
Gls2
Thap2
Kit
Tspan2
Atp2c1
C430048L16Rik
Nsdhl
B630005N14Rik
Nipa2
Pcsk1
Pkia
Pank3
Slc19a2
Zdhhc2
Lgals8
Parm1
Rcbtb1
Dhx40
Pam
Rasgef1c
Slc25a36
Abca5
Serpini1
Idh1
Tmtc3
Atp13a3
Plekhh1
Ldlr
Papolg
Slc12a2
Sept2
Dennd5b
Rnf170
Wdfy1
Pdgfra
Adcyap1
Sim1
Pgr15l
Cartpt
Crabp1
Bdnf
C130071C03Rik
Espn
Ghsr

Supplementary Figure 8. Net methylation changes over promoters and gene bodies for genes downregulated in *Dnmt3a*$^{\Delta/D329A}$ adult hypothalamus. Methylation differences between *Dnmt3a*$^{+/+}$ and *Dnmt3a*$^{\Delta/D392A}$ were quantified over promoters and gene bodies. Promoter probes within 1kb distance were merged. Clustering was performed using the Euclidean complete method. $n(+/+, \Delta/D329A) = 3$.

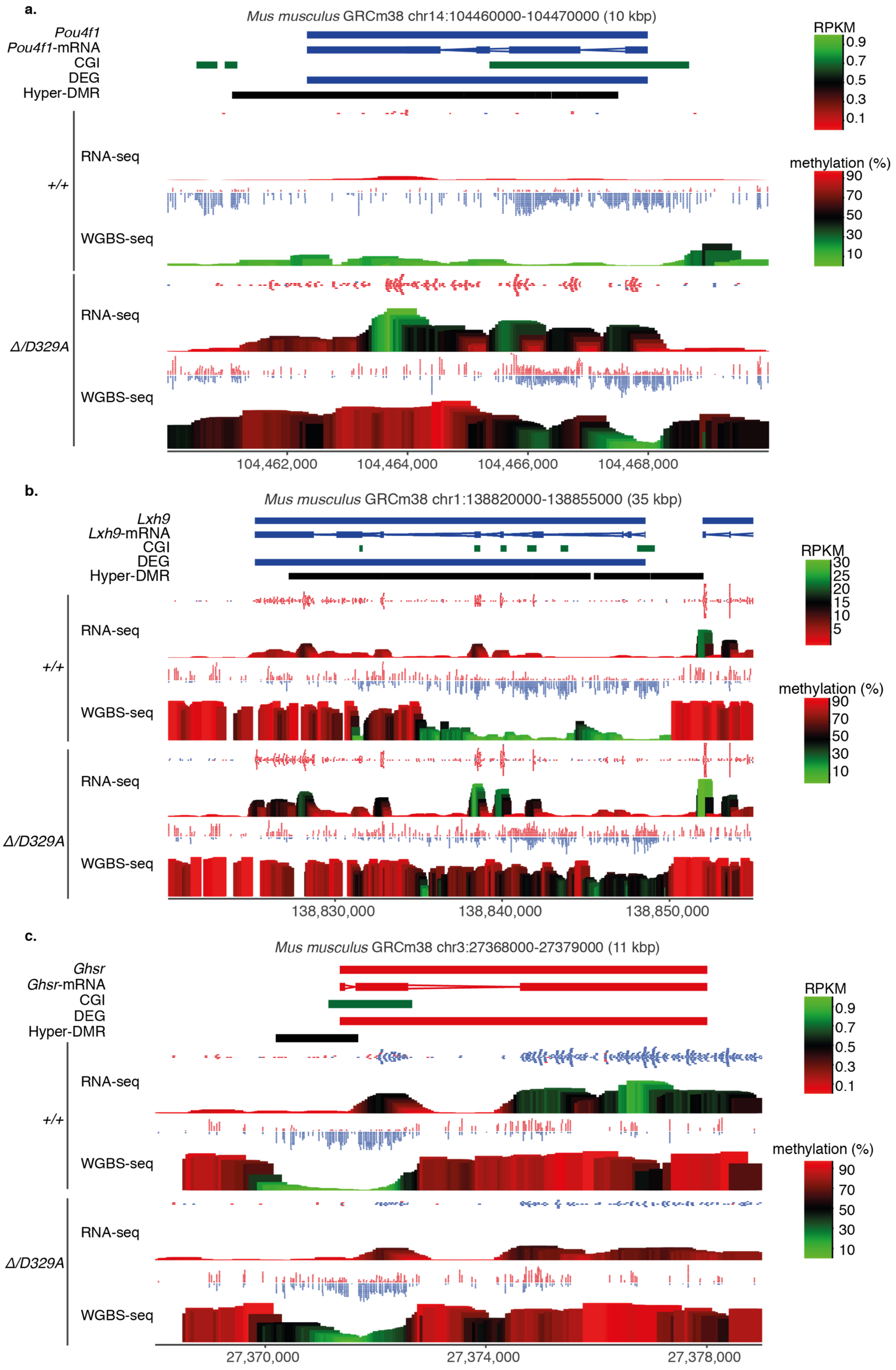
a.
Mus musculus GRCm38 chr14:104460000-104470000 (10 kbp)
Pou4f1
Pou4f1-mRNA
CGI
DEG
Hyper-DMR
+/+
RNA-seq
WGBS-seq
Δ/D329A
RNA-seq
WGBS-seq
104,462,000
104,464,000
104,466,000
104,468,000
RPKM
0.9
0.7
0.5
0.3
0.1
methylation (%)
90
70
50
30
10
b.
Mus musculus GRCm38 chr1:138820000-138855000 (35 kbp)
Lxh9
Lxh9-mRNA
CGI
DEG
Hyper-DMR
+/+
RNA-seq
WGBS-seq
Δ/D329A
RNA-seq
WGBS-seq
138,830,000
138,840,000
138,850,000
RPKM
30
25
20
15
10
5
methylation (%)
90
70
50
30
10
c.
Mus musculus GRCm38 chr3:27368000-27379000 (11 kbp)
Ghsr
Ghsr-mRNA
CGI
DEG
Hyper-DMR
+/+
RNA-seq
WGBS-seq
Δ/D329A
RNA-seq
WGBS-seq
27,370,000
27,374,000
27,378,000
RPKM
0.9
0.7
0.5
0.3
0.1
methylation (%)
90
70
50
30
10

Supplementary Figure 9. Genome browser views of representative regions that show altered gene expression and differential methylation in *Dnmt3a*$^{\Delta/D392A}$ hypothalamus.
a-c, Genome browser views from Seqmonk of the (**a**) *Pou4f1* locus that is de-repressed and exhibits methylation gain over the gene body, (**b**) *Lhx9* locus that is upregulated and exhibits methylation gain over both gene body and promoter region, (**c**) *Ghsr* locus that is downregulated and exhibits methylation gain over the promoter region. Each coloured block represents a 500bp tile with 50bp step. Raw sequencing reads are shown. For raw reads, gene and mRNA tracks, the colour indicates direction, where red is a forward strand and blue is a reverse strand. Libraries were prepared using an opposite-strand specific kit. For methylation red means a methylated call and blue means an unmethylated call, each point indicating an individual C in CpG context. Abbreviations: CGI: CpG island, Hyper-DMR: hypermethylated region, DEG: differentially expressed gene. *n*(+/+) = 3, *n*(+/*D329A*) = 2.

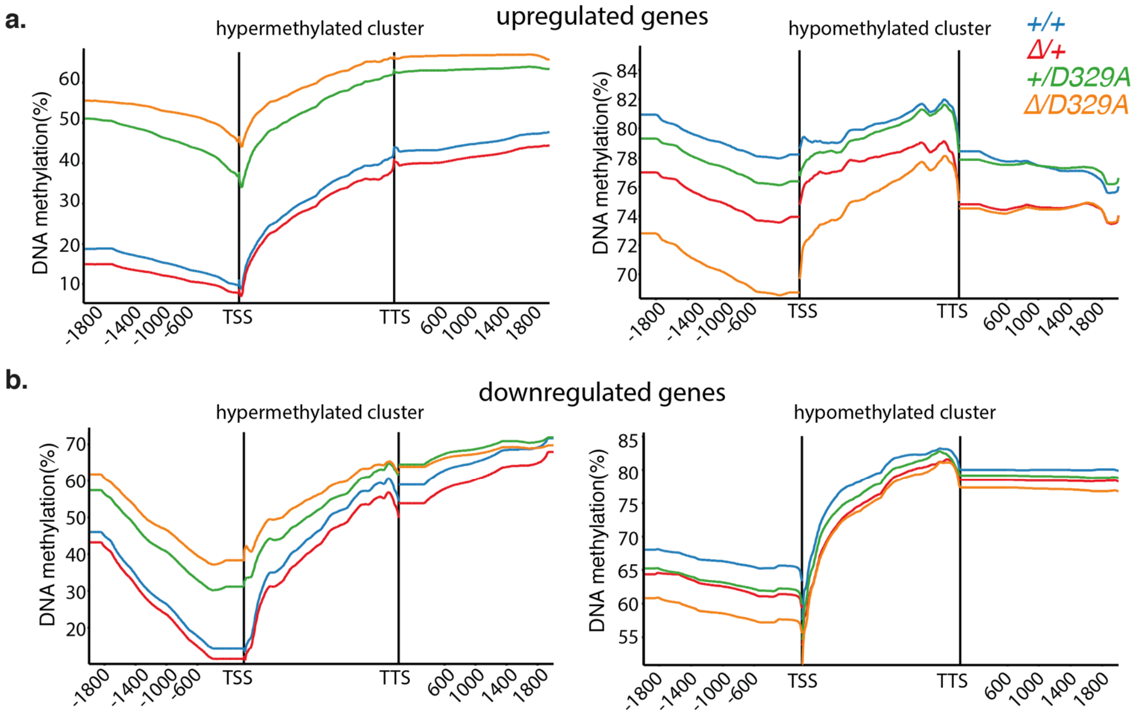

Supplementary Figure 10. Trendplots showing DNA methylation over genes differentially expressed in adult hypothalamus with methylation changes in their promoter region.
Aggregated methylation levels over differentially expressed gene clusters showing hyper- and hypo- methylated promoters, marked in Supplementary Fig. 7 and 8. TSS: transcription start site, TTS: transcription termination site. Distance over gene is relative. *n*(upregulated genes, hypermethylated promoters) = 24, *n*(upregulated genes, hypomethylated promoters) = 25, *n*(downregulated genes, hypermethylated promoters) = 10, *n*(upregulated genes, hypomethylated promoters) = 10. *n*(methylation datasets, +/+, *Δ*/+, *Δ/D329A*) = 3, *n*(+/*D329A*) = 2.

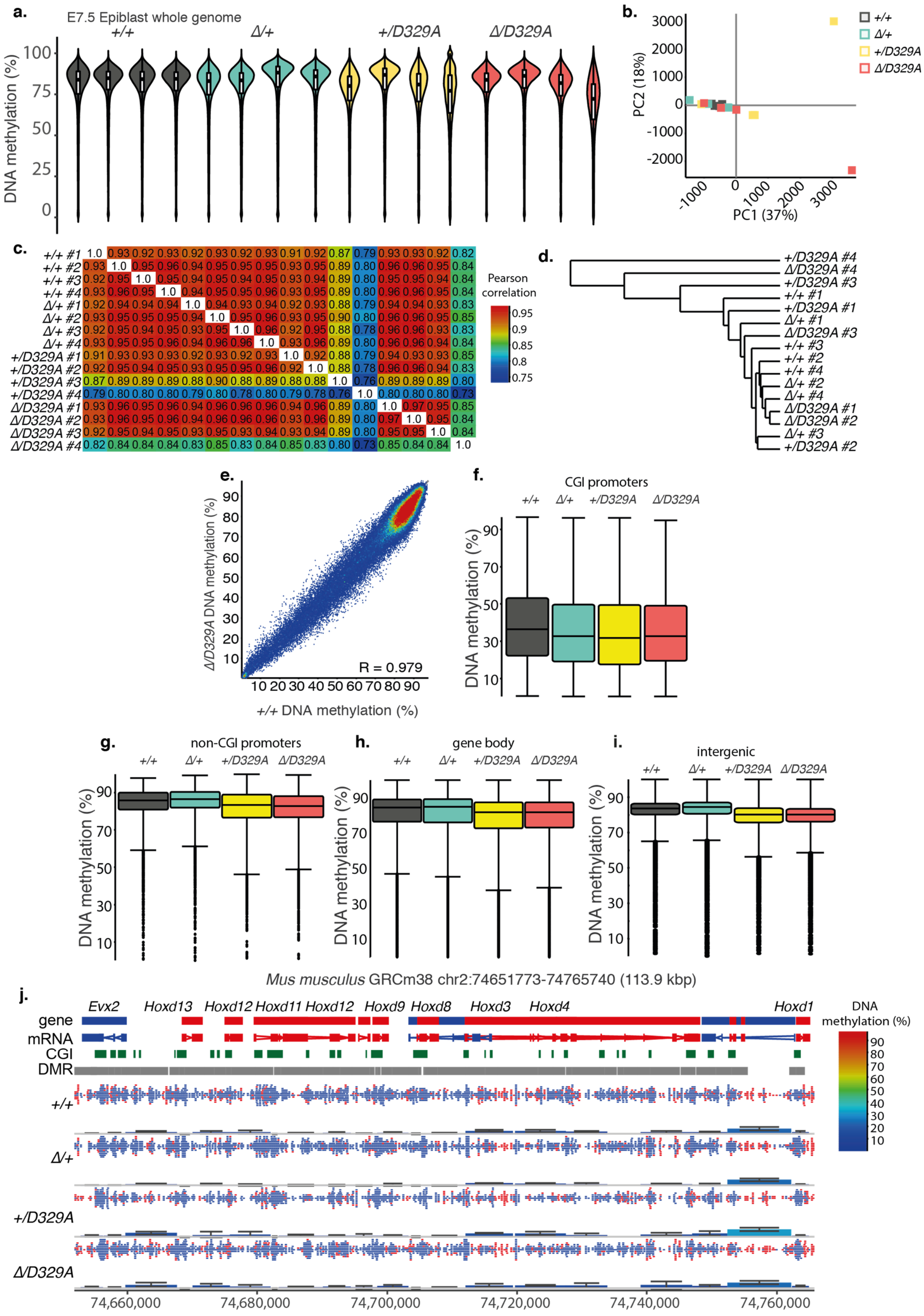
a.
E7.5 Epiblast whole genome
+/+
Δ/+
+/D329A
Δ/D329A
DNA methylation (%)
b.
PC2 (18%)
PC1 (37%)
c.
Pearson correlation
d.
e.
Δ/D329A DNA methylation (%)
+/+ DNA methylation (%)
R = 0.979
f.
CGI promoters
g.
non-CGI promoters
h.
gene body
i.
intergenic
Mus musculus GRCm38 chr2:74651773-74765740 (113.9 kbp)
j.
gene
mRNA
CGI
DMR
Evx2
Hoxd13
Hoxd12
Hoxd11
Hoxd12
Hoxd9
Hoxd8
Hoxd3
Hoxd4
Hoxd1
DNA methylation (%)
74,660,000
74,680,000
74,700,000
74,720,000
74,740,000
74,760,000

Supplementary Figure 11. DNA methylation in E7.5 epiblast.
a, Beanplots indicating whole genome methylation levels in individual E7.5 epiblasts of different genotypes. Tiles of 300-CpG positions were used. Boxplots show median value and 25-75th percentiles, whiskers show lowest and highest observation, excluding outliers. Each plot represents the data from an individual mouse. Variation within individuals could be attributable to developmental timing. Raw data is provided in Source Data.
b, PCA plot showing clustering of the majority of the individual E7.5 epiblasts together. The two outliers arise from two lowest methylation samples observed in (**a**).
c, A table showing pairwise Pearson correlation values for individual sample pairs, where value of 1.0 is an ideal correlation.
d, A data similarity tree indicating Pearson correlation distances between individual samples.
e, Scatterplot showing correlation between methylation levels of individual tiles between *Dnmt3a*$^{+/+}$ and *Dnmt3a*$^{\Delta/D329A}$ E7.5 epiblasts.
f-i, DNA methylation levels in E7.5 epiblast quantified across (**f**) CGI promoters, (**g**) non-CGI promoters, (**h**) gene bodies (excluding promoters), and (**i**) intergenic regions (excluding gene bodies and promoters). Boxplots show median value and 25-75th percentiles, whiskers show lowest and highest observation, outliers are marked by individual dots. Raw data are provided in Source Data.
j, Genome browser view of the *Hoxd* gene cluster in E7.5 epiblast. The track for each genotype displays methylation calls for individual CpGs, where methylated positions are red and unmethylated positions are blue (above), as well as quantification of 300-CpG tiles (below). CGI: CpG island, DMR: hypermethylated region in adult tissues. For gene and mRNA tracks, the colour indicates direction, where red is a forward strand and blue is a reverse strand. Error bars indicate standard deviation.
In **a-j**, Tiles of 300-CpG positions; n = 4 for each genotype.

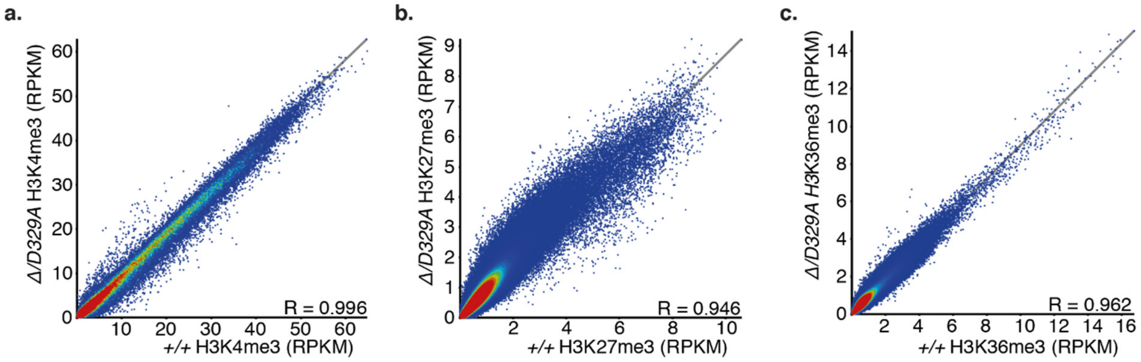

Supplementary Figure 12. Chromatin-immunoprecipitation of H3K4me3, H3K27me3 and H3K36me3 in adult hypothalamus.
a-c, Scatterplots showing correlation for (**a**) H3K4me3; (**b**) H3K27me3; (**c**) H3K36me3 enrichments between *Dnmt3a*$^{+/+}$ and *Dnmt3a*$^{\Delta/D329A}$ hypothalamus. n = 3 each genotype. Tiles of 2kb with a step of 1kb were used for analyses.

D329A allele transmission	Matings	Litters born	Pups	Weaned
Maternal	19	2	4	0
Paternal	23	19	158	86

Supplementary Table 1. Numbers of matings, litters, pups born and weaned when the *Dnmt3a*^{D329A} allele was transmitted paternally or maternally. Results of crossing *Dnmt3a*$^{+/D329A}$ carriers with C57BL/6Babr.

Confirmed pregnant	Pups	Weaned
9	29	13

Supplementary Table 2. Numbers of pregnancies, pups cross-fostered and weaned when *Dnmt3a*$^{+/D329A}$ females in late pregnancy were subjected to caesarean section and pups fostered to CD1 mothers.

Male	10 days	4 weeks	6 weeks	8 weeks	10 weeks	12 weeks
+/+	19	19	18	19	19	18
Δ/+	12	12	12	12	12	12
+/D329A	7	7	8	8	7	7
Δ/D329A	12	12	12	13	13	11
Female	10 days	4 weeks	6 weeks	8 weeks	10 weeks	12 weeks
+/+	7	7	8	8	8	8
Δ/+	11	11	13	13	13	13
+/D329A	4	4	5	5	5	5
Δ/D329A	8	8	8	8	8	8

Supplementary Table 3. Numbers of individual mice at indicated ages used (*n* value) to generate body weight curve plots (Figure 1c).
