# Supplementary material for: A DNMT3A PWWP mutation leads to methylation of bivalent chromatin and growth retardation in mice
Source: Nat Commun. 2019 Apr 23;10:1884. doi: 10.1038/s41467-019-09713-w (PMC6478690; doi:10.1038/s41467-019-09713-w)
Supplement: Supplementary file 3 — Reporting Summary [file 41467_2019_9713_MOESM3_ESM.pdf]

nature research

Corresponding author(s): Gavin Kelsey

Reporting Summary

Nature Research wishes to improve the reproducibility of the work that we publish. This form provides structure for consistency and transparency in reporting. For further information on Nature Research policies, see Authors & Referees and the Editorial Policy Checklist.

Statistical parameters

When statistical analyses are reported, confirm that the following items are present in the relevant location (e.g. figure legend, table legend, main text, or Methods section).

n/a	Confirmed	
☐	☒	The exact sample size (*n*) for each experimental group/condition, given as a discrete number and unit of measurement
☐	☒	An indication of whether measurements were taken from distinct samples or whether the same sample was measured repeatedly
☐	☒	The statistical test(s) used AND whether they are one- or two-sided<br>*Only common tests should be described solely by name; describe more complex techniques in the Methods section.*
☒	☐	A description of all covariates tested
☐	☒	A description of any assumptions or corrections, such as tests of normality and adjustment for multiple comparisons
☐	☒	A full description of the statistics including central tendency (e.g. means) or other basic estimates (e.g. regression coefficient) AND variation (e.g. standard deviation) or associated estimates of uncertainty (e.g. confidence intervals)
☐	☒	For null hypothesis testing, the test statistic (e.g. *F*, *t*, *r*) with confidence intervals, effect sizes, degrees of freedom and *P* value noted<br>*Give P values as exact values whenever suitable.*
☒	☐	For Bayesian analysis, information on the choice of priors and Markov chain Monte Carlo settings
☐	☒	For hierarchical and complex designs, identification of the appropriate level for tests and full reporting of outcomes
☒	☐	Estimates of effect sizes (e.g. Cohen's *d*, Pearson's *r*), indicating how they were calculated
☐	☒	Clearly defined error bars<br>*State explicitly what error bars represent (e.g. SD, SE, CI)*

Our web collection on statistics for biologists may be useful.

Software and code

Policy information about availability of computer code

Data collection	All software and code used is open-source and publicly available. Data was processed using TrimGalore 0.5.0, Bismark 0.19.1, Bowtie 1.2.2, Hisat2 2.1.0.
Data analysis	All software and code used is open-source and publicly available. The analysis was done using Seqmonk 1.42.0 and R Statistical Software 3.4.1.

For manuscripts utilizing custom algorithms or software that are central to the research but not yet described in published literature, software must be made available to editors/reviewers upon request. We strongly encourage code deposition in a community repository (e.g. GitHub). See the Nature Research guidelines for submitting code & software for further information.

Data

Policy information about availability of data

All manuscripts must include a data availability statement. This statement should provide the following information, where applicable:

- Accession codes, unique identifiers, or web links for publicly available datasets
- A list of figures that have associated raw data
- A description of any restrictions on data availability

Sequence data that support the findings of this study have been deposited in Gene Expression Omnibus with the primary accession code GSE117728.
Raw data for Figures 1c, 2a, 3a, 4a-b, 6e-i, 8b-c and Supplementary Figures 3, 4a, 4c-d, 5a-b, 11a, 11f-i is provided in Source Data. Raw data for Figures 2c-d, 5b is provided in Supplementary Data.

Field-specific reporting

Please select the best fit for your research. If you are not sure, read the appropriate sections before making your selection.

☒ Life sciences ☐ Behavioural & social sciences ☐ Ecological, evolutionary & environmental sciences

For a reference copy of the document with all sections, see nature.com/authors/policies/ReportingSummary-flat.pdf

Life sciences study design

All studies must disclose on these points even when the disclosure is negative.

Sample size	No statistical method was used to predetermine sample size.
Data exclusions	One hypothalamus adult Dnmt3a_Del/D329A RNA-seq sample was excluded from the analysis. Initial quality control analysis showed it was enriched for reads of 30bp length, had highly skewed mean GC content and contained approximately 50% less data than the next lowest coverage sample within the cohort. Subsequently, hierarchical clustering showed it branched off early and was dissimilar to any other samples, and had gene expression range which lacked values falling within range of -4 to -2 log2(RPKM), unlike other 9 samples analysed.
Replication	All experiments were done in biological replicates, which are reported in the Figures, Legends and Methods.
Randomization	Experimental groups were allocated based on tissue type, genotype and age of mice, thus it was not completely randomised. Otherwise, all tissues were collected in litters which had a random number of mice of each genotype. Samples were selected from a range of different litters based solely on genotype and age.
Blinding	The investigators were not blinded to the group allocation during experiments or outcome assessment because genotypes of animals had to be known in prior.

Reporting for specific materials, systems and methods

Materials & experimental systems

n/a	Involved in the study	
☒	☐	Unique biological materials
☐	☒	Antibodies
☒	☐	Eukaryotic cell lines
☒	☐	Palaeontology
☐	☒	Animals and other organisms
☒	☐	Human research participants

Methods

n/a	Involved in the study	
☐	☒	ChIP-seq
☒	☐	Flow cytometry
☒	☐	MRI-based neuroimaging

Antibodies

Antibodies used	anti-H3K27me3 (Millipore 07-449)<br>anti-H3K36 (Diagenode C15410192)<br>anti-H3K4me3 (Diagenode C15410003)
Validation	These antibodies are widely used for ChIP-seq and have been validated by their respective source companies.

Animals and other organisms

Policy information about studies involving animals; ARRIVE guidelines recommended for reporting animal research

Laboratory animals	Mouse (Mus musculus, strain C57BL/6Babr) tissues collected from both males and females animals at ages E7.5, P1, P25 and ~14 weeks were used in this study.
Wild animals	Study did not involve wild animals.
Field-collected samples	Study did not involve field-collected samples.

ChIP-seq

Data deposition

☒ Confirm that both raw and final processed data have been deposited in a public database such as GEO.

☒ Confirm that you have deposited or provided access to graph files (e.g. BED files) for the called peaks.

Data access links
May remain private before publication.

Sequence data that support the findings of this study have been deposited in Gene Expression Omnibus with the primary accession code GSE117728.

Files in database submission

Adult_ht_ChIP_K4_++_1
Adult_ht_ChIP_K4_++_2
Adult_ht_ChIP_K4_++_3
Adult_ht_ChIP_K4_del-mut_1
Adult_ht_ChIP_K4_del-mut_2
Adult_ht_ChIP_K4_del-mut_3
Adult_ht_ChIP_K27_++_1
Adult_ht_ChIP_K27_++_2
Adult_ht_ChIP_K27_++_3
Adult_ht_ChIP_K27_del-mut_1
Adult_ht_ChIP_K27_del-mut_2
Adult_ht_ChIP_K27_del-mut_3
Adult_ht_ChIP_K36_++_1
Adult_ht_ChIP_K36_++_2
Adult_ht_ChIP_K36_++_3
Adult_ht_ChIP_K36_del-mut_1
Adult_ht_ChIP_K36_del-mut_2
Adult_ht_ChIP_K36_del-mut_3
Adult_ht-ChIP_input_++_1
Adult_ht-ChIP_input_++_2
Adult_ht-ChIP_input_++_3
Adult_ht_ChIP_input_del-mut_1
Adult_ht_ChIP_input_del-mut_2
Adult_ht_ChIP_input_del-mut_3

Genome browser session
(e.g. UCSC)

http://epigenomegateway.wustl.edu/browser/?genome=mm10&session=klvBjEz5z5&statusId=1188949108

Methodology

Replicates

Three biological replicates were used per each genotype. Every individual sample was then used for H3K4me3, H4K27me3, H3K36me3 and input ChIP-seq library generation.
Replicate agreement:
H3K4me3 +/+ : 0.9923281, 0.9933433, 0.99309784;
H3K4me3 del/D329A : 0.984117, 0.9881814, 0.9893899;
H3K27me3 +/+ : 0.8889338, 0.8922302, 0.87033165;
H3K27me3 del/D329A : 0.8887717, 0.867342, 0.89176285;
H3K36me3 +/+ : 0.9215261, 0.91227573, 0.91026247;
H3K36me3 del/D329A : 0.89590716, 0.8956215, 0.8923797;
input +/+ : 0.66748625, 0.6788064, 0.72019804;
input del/D329A : 0.7562018, 0.7471351, 0.72750074.

Sequencing depth

All libraries were sequenced in 75bp single-end mode.
Adult_ht_ChIP_K4_++_1 Total: 26065430 Uniquely mapped: 15811942;
Adult_ht_ChIP_K4_++_2 Total: 24742605 Uniquely mapped: 15035465;
Adult_ht_ChIP_K4_++_3 Total: 25346843 Uniquely mapped: 15223889;
Adult_ht_ChIP_K4_del-mut_1 Total:25531851 Uniquely mapped: 15944967;
Adult_ht_ChIP_K4_del-mut_2 Total: 20052247 Uniquely mapped: 12676361;
Adult_ht_ChIP_K4_del-mut_3 Total: 28877523 Uniquely mapped: 15493430;
Adult_ht_ChIP_K27_++_1 Total: 23941847 Uniquely mapped: 15891090;
Adult_ht_ChIP_K27_++_2 Total: 25697305 Uniquely mapped: 16654128;

Adult_ht_ChIP_K27_++_3 Total: 30991066 Uniquely mapped: 20169667;
Adult_ht_ChIP_K27_del-mut_1 Total:23927549 Uniquely mapped: 14874941;
Adult_ht_ChIP_K27_del-mut_2 Total: 39804568 Uniquely mapped: 26178087;
Adult_ht_ChIP_K27_del-mut_3 Total: 30309992 Uniquely mapped: 19697272;
Adult_ht_ChIP_K36_++_1 Total: 32880597 Uniquely mapped: 22850000;
Adult_ht_ChIP_K36_++_2 Total: 29471102 Uniquely mapped: 21092107;
Adult_ht_ChIP_K36_++_3 Total: 28734886 Uniquely mapped: 19107197;
Adult_ht_ChIP_K36_del-mut_1 Total: 29937232 Uniquely mapped: 20537472;
Adult_ht_ChIP_K36_del-mut_2 Total: 30238060 Uniquely mapped: 20345215;
Adult_ht_ChIP_K36_del-mut_3 Total: 28528176 Uniquely mapped: 19056381;
Adult_ht-ChIP_input_++_1 Total: 18726261 Uniquely mapped: 14708936;
Adult_ht-ChIP_input_++_2 Total: 28176825 Uniquely mapped: 21579336;
Adult_ht-ChIP_input_++_3 Total: 30719847 Uniquely mapped: 23890358;
Adult_ht_ChIP_input_del-mut_1 Total: 39855286 Uniquely mapped: 30207874;
Adult_ht_ChIP_input_del-mut_2 Total: 32097044 Uniquely mapped: 24772961;
Adult_ht_ChIP_input_del-mut_3 Total: 28336929 Uniquely mapped: 21697211.

Antibodies

anti-H3K27me3 (Millipore 07-449)
anti-H3K36 (Diagenode C15410192)
anti-H3K4me3 (Diagenode C15410003)

Peak calling parameters

Generally the analysis was done using enrichment over genome tiles. When needed, peaks were called using MACS peak caller embeded within Seqmonk 1.42.0 software with default parameters, using a significant threshold of p<0.00001, a fragment size of 1kb, and input controls as a reference.

Data quality

Hierarchical clustering and replicate correlations were used to assess data quality.

Software

The analysis was done using Seqmonk 1.42.0.
