# Supplementary material for: A DNMT3A PWWP mutation leads to methylation of bivalent chromatin and growth retardation in mice
Source: Nat Commun. 2019 Apr 23;10:1884. doi: 10.1038/s41467-019-09713-w (PMC6478690; doi:10.1038/s41467-019-09713-w)
Supplement: Supplementary file 4 — Description of Additional Supplementary Files [file 41467_2019_9713_MOESM4_ESM.pdf]

Description of Additional Supplementary Files

File Name: Supplementary Data 1
Description: Gene expression log2(RPKM) values for genes showing differential expression (DESeq p<0.01) between Dnmt3a+/+ and Dnmt3aΔ/D329A in adult mice hypothalamus.

File Name: Supplementary Data 2
Description: Gene ontology analysis (DAVID 6.7) of differentially expressed genes in adult mice hypothalamus.

File Name: Supplementary Data 3
Description: Domains showing differential methylation between Dnmt3a+/+ and Dnmt3aΔ/D329A in adult mice hypothalamus.

File Name: Supplementary Data 4
Description: Domains showing differential methylation between Dnmt3aΔ/+ and Dnmt3aΔ/D329A in adult mice hypothalamus.

File Name: Supplementary Data 5
Description: Domains showing differential methylation between Dnmt3a+/+ and Dnmt3aΔ/+ in adult mice hypothalamus.

File Name: Supplementary Data 6
Description: Domains showing differential methylation between Dnmt3a+/+ and Dnmt3aΔ/D329A in adult mice pituitary.

File Name: Supplementary Data 7
Description: Domains showing differential methylation between Dnmt3aΔ/+ and Dnmt3aΔ/D329A in adult mice pituitary.

File Name: Supplementary Data 8
Description: Domains showing differential methylation between Dnmt3a+/+ and Dnmt3aΔ/D329A in adult mice liver.

File Name: Supplementary Data 9
Description: Domains showing differential methylation between Dnmt3aΔ/+ and Dnmt3aΔ/D329A in adult mice liver.

File Name: Supplementary Data 10
Description: Domains showing differential methylation between Dnmt3a+/+ and Dnmt3aΔ/+ in E7.5 Epiblast.

File Name: Supplementary Data 11
Description: Domains showing differential methylation between Dnmt3a+/+ and Dnmt3aΔ/D329A in E7.5 Epiblast.

File Name: Supplementary Data 12
Description: Locations and methylation values of DNA canyons previosuly described by Jeong et al.60 in adult mouse hypothalamus.

File Name: Supplementary Data 13
Description: Gene ontology analysis (DAVID 6.8) of differentially methylated regions in adult mice hypothalamus.

File Name: Supplementary Data 14
Description: Locations of random probes used as representative of the whole genome.
