# Supplementary material for: A DNMT3A PWWP mutation leads to methylation of bivalent chromatin and growth retardation in mice
Source: Nat Commun. 2019 Apr 23;10:1884. doi: 10.1038/s41467-019-09713-w (PMC6478690; doi:10.1038/s41467-019-09713-w)
Supplement: Supplementary file 2 — Peer Review File [file 41467_2019_9713_MOESM2_ESM.pdf]

Editorial Note: This manuscript has been previously reviewed at another journal that is not operating a transparent peer review scheme. This document only contains reviewer comments and rebuttal letters for versions considered at *Nature Communications.*

Reviewers' comments:

Reviewer #1 (Remarks to the Author):

Using mouse genetics, Sendzikaite et al. have studied the role of K36me2/3 binding of the DNMT3A PWWP domain. They report an interesting gain of function effect of the mutation leading to an unexpected hypermethylation of PRC2 domains. The authors report a novel and potentially very interesting observation that could make an important contribution to the understanding of the biological role of DNMT3A. The paper is very interesting and the experiments were conducted technically at highest standards. It reports very important results that will have a big impact on the entire field.

Major comments

1) The description of the state of the art regarding the PWWP domain on p. 4must be improved in several instances.
Line 62: ref 44 must be cited here. It may be of relevance for readers that while ref 44 in the initial discovery found H3K36me3 binding on peptide arrays and be equilibrium peptide binding, later papers based on peptide arrays repeatedly showed binding to H3K36me2/3 (PMID: 21884582, PMID: 25301795, PMID: 28946896).
Line 66-67: The description of the negative data for DNMT3B is a bit misleading. The biochemical data in 44 do not show K36me binding by the DNMT3B PWWP domain. The structural data in 40 show a complex for DNMT3B PWWP domain, but not for DNMT3A. In both cases the negative data may be caused by technical issues, so the more unified view is that both domain apparently can bind H3K36me2/3.
p. 4, line 76-80: This part needs to be moved to the introduction. Ref. 48 and 44 point to the same paper.
Line 76: ref 44 (48) also provides peptide binding data, not only peptide array.

2) I find many data currently shown in Supplement very interesting (particularly S7, S12, S14 and S15). In the light of the relaxed space constraints of Nat. Comm., I would strongly encourage authors and editors to take efforts moving as many of the data into the main article to maximize their impact and facilitate reading of the paper.

3) The E7.5 data are not fully convincing and consistent with the rest of the paper. For example, the data in S13A are not in agreement with the previous statement, that hypomethylation in D329A is caused by haploinsufficiency, because there is mutation dependent hypomethylation. The authors should check and report where hypomethylated tiles in this data set are enriched. This will help to understand the role of DNMT3A in early development.

4) Fig. 5A is confusing and difficult to read. The scatter plot likely is not the best way to show these data. Also, it is not explained what the pie chart displays. Same true for S14g.

5) The authors argue in the direction of a recruitment of the mutant DNMT3A to K27me3 regions. This model is against a passive model proposed recently by ref. 27. I am not fully convinced by the evidence for this. PMID: 30468428 is reporting methylation at K27me3 regions after overexpression of wildtype DNMT3B, how can this be explained? One model in my view fitting all data would be a (weak) intrinsic targeting of DNMT3A (and 3B) to K27me3 regions, normally overruled by the strong recruitment to H3K36me3 (both marks are largely antagonistic). If the K36me3 binding is lost, only K27me3 targeting remains. But this is not caused by the mutation. If one overexpressed K36me3 targeting is saturated and then K27me3 targeting also occurs.

6) p 13, line 310+311: recently several papers reported binding of PRC2 to CpG methylated DNA. This sentence should be updated.

7) p. 13, line 312: There were effects observed in E7.5, but they were not properly analyzed. This sentence at least should be removed.

8) p. 13, line 328-330: I do not agree with this sentence. See point 5.

Reviewer #2 (Remarks to the Author):

The revised manuscript is improved, and my previous questions were addressed to some extent, although a few responses are still not quite convincing.

For example, the linkages among several main findings of the paper are still lacking. The additional analysis comparing H3K27me3 and DNA hypermethylation in mutants provides helpful supporting evidence but is still a correlation rather than direct proof of Dnmt3a recruitment. Also this again does not fully exclude the possibility that this is because these regions are hypomethylated from the beginning. Second, I am still not fully convinced that differentially expressed genes expressed at basal levels (FPKM < 1 in both WT and mutant) are functional. This is not impossible but certainly requires functional evidence. Statistics are helpful but are known to be mis-leading sometimes. Finally, it is not clear to me why the relationship between DNA hypermethylation and gene expression is a “subsidiary question”, given the obvious linkage between DNA methylation and gene expression.

I understand some of these mechanistic experiments are challenging within the revision period. Yet the response letter appeared to be poorly written. The related conclusions above should be at least tuned down, before it can be considered for publication in NC.

NCOMMS-18-37103-T Response to Reviewers

Alterations to the manuscript text are highlighted in yellow.

Reviewer #1 (Remarks to the Author):

Using mouse genetics, Sendzikaite et al. have studied the role of K36me2/3 binding of the DNMT3A PWWP domain. They report an interesting gain of function effect of the mutation leading to an unexpected hypermethylation of PRC2 domains. The authors report a novel and potentially very interesting observation that could make an important contribution to the understanding of the biological role of DNMT3A. The paper is very interesting and the experiments were conducted technically at highest standards. It reports very important results that will have a big impact on the entire field.

>> We are grateful to the reviewer for his/her very positive assessment of the new manuscript.

Major comments

1) The description of the state of the art regarding the PWWP domain on p. 4 must be improved in several instances.
Line 62: ref 44 must be cited here.

>> The paragraph (now lines 61-82) on the PWWP domain has been considerably revised and extended and we hope it is now a more complete description of the state of the art. The Dhayalan et al. 2010 citation (ref. 44) was added where the reviewer suggested.

It may be of relevance for readers that while ref 44 in the initial discovery found H3K36me3 binding on peptide arrays and be equilibrium peptide binding, later papers based on peptide arrays repeatedly showed binding to H3K36me2/3 (PMID: 21884582, PMID: 25301795, PMID: 28946896).

>> As recommended, in the extended paragraph we have included a statement to this effect (now lines 67-69):

"Biochemical studies, including chromatin precipitation and peptide arrays, have reproducibly demonstrated that the PWWP domain of DNMT3A interacts exclusively with H3K36me2/3 (ref. 44-47)."

Line 66-67: The description of the negative data for DNMT3B is a bit misleading. The biochemical data in 44 do not show K36me binding by the DNMT3B PWWP domain. The structural data in 40 show a complex for DNMT3B PWWP domain, but not for DNMT3A. In both cases the negative data may be caused by technical issues, so the more unified view is that both domain apparently can bind H3K36me2/3.

>> The text in this same paragraph (now lines 73-76) has been updated to include the negative data in relation to the study by Baubec et al. 2015, which specifically indicates that it was DNMT3B, but not DNMT3A, responsible for gene body methylation in mouse embryonic stem cells.

p. 4, line 76-80: This part needs to be moved to the introduction. Ref. 48 and 44 point to the same paper. Line 76: ref 44 (48) also provides peptide binding data, not only peptide array.

>> The statement has been removed from the opening of the Results section and incorporated into the Introduction (now lines 67-73). The reference duplication has been removed.

2) I find many data currently shown in Supplement very interesting (particularly S7, S12, S14 and S15). In the light of the relaxed space constraints of Nat. Comm., I would strongly encourage authors and editors to take efforts moving as many of the data into the main article to maximize their impact and facilitate reading of the paper.

>> This was a very useful recommendation and we are happy to move data from Supplementary Figures and into Main Figures in order to improve clarity. Therefore:

1. We have incorporated the scatterplots from Fig. S7c,d (now Fig. S6) into Fig. 2 (new parts e, f). By presenting all the pairwise genotype comparisons, this helps demonstrate the effect of haploinsufficiency from the effect of the D329A mutation.
2. We present a slightly simplified version of Fig. S12 as new main Fig. 3, which illustrates the presence of hypermethylated regions in liver and pituitary and the extent of the overlap with those in hypothalamus.
3. We have moved scatterplots for H3K4me3, H3K27me3 and H3K36me3 ChIP-seq data from Fig. S14d-f (now Fig. S13) into a revised Fig. 6 (parts a-c). This illustrates the distribution of hyper- and hypo- DMRs over the distributions of H3K4me3, H3K27me3 and H3K36me3 enrichment. We also moved Fig. S14g,h (now Fig. S13) into revised Fig. 6g,h. The boxplots display DNA methylation levels over normally hypomethylated domains showing low- or high-H3K4me and H3K36me3 enrichment.
4. We have made Fig. S15 into a new main Fig. 7. This provides examples of strongly de-repressed genes over which patterns of DNA methylation and histone modifications are altered as a result of the D329A mutation.

3) The E7.5 data are not fully convincing and consistent with the rest of the paper. For example, the data in S13A are not in agreement with the previous statement, that hypomethylation in D329A is caused by haploinsufficiency, because there is mutation dependent hypomethylation. The authors

should check and report where hypomethylated tiles in this data set are enriched. This will help to understand the role of DNMT3A in early development.

>> The main finding we wished to take from the E7.5 DNA methylation data was that the ectopic methylation (hypermethylation) observed in adult tissues does not originate at this developmental stage. On the reviewer's suggestion, we have looked in more detail at the E7.5 data. We are happy to agree with his/her interpretation that that there is mutation-dependent hypomethylation, which appears to be distinct from wild-type DNMT3A haploinsufficiency. Our data suggests that wild-type DNMT3A plays a role at CGI methylation in early development, as that is where we observe consistent allele dosage dependent hypomethylation. Meanwhile, mutant embryos appear to have increased variability, which suggests that presence of the mutant protein causes a delay in global methylation establishment or developmental delay of unknown extent. Importantly, embryos do not appear to cluster by genotype based on their methylation patterns, and we do not identify many statistically significant DMRs. Our interpretation is that this indicates that dysfunctionality of the mutant protein is stochastic rather than targeted. Accordingly, we have revised the paragraph in the Results to include these further analyses (now lines 191-205), and discuss them in a revised Discussion paragraph (lines 300-311). We have also updated Supplementary Fig. S12 to include measures of how the methylation datasets from the four genotypes cluster/correlate (Fig. S12b-d) and differ among genomic features (Fig. S12f-i).

4) Fig. 5A is confusing and difficult to read. The scatter plot likely is not the best way to show these data. Also, it is not explained what the pie chart displays. Same true for S14g.

>> On the reviewer's advice, we have removed Fig. 5A and replaced it with a stacked barchart (now Fig. 6d) that demonstrates the proportions of different histone mark peaks falling over hyper-, hypo- DMRs, and random probes (as genome representative). We hope this presents more information with greater clarity. This new plot also makes the former Fig. S14g redundant, which has now been removed.

5) The authors argue in the direction of a recruitment of the mutant DNMT3A to K27me3 regions. This model is against a passive model proposed recently by ref. 27. I am not fully convinced by the evidence for this. PMID: 30468428 is reporting methylation at K27me3 regions after overexpression of wildtype DNMT3B, how can this be explained? One model in my view fitting all data would be a (weak) intrinsic targeting of DNMT3A (and 3B) to K27me3 regions, normally overruled by the strong recruitment to H3K36me3 (both marks are largely antagonistic). If the K36me3 binding is lost, only K27me3 targeting remains. But this is not caused by the mutation. If one overexpressed K36me3 targeting is saturated and then K27me3 targeting also occurs.

>> We still favour our model for an enhanced recruitment to domains of H3K27me3, rather than relocalisation to any hypomethylated domains, because it is more compatible with our own observations from our data, the developmental profile of the ectopic methylation, and the known association of the DNMT3A1 isoform with H3K27me3-marked CGI shores. Nevertheless, we agree with the reviewer that it is important to discuss alternative explanations, and we do now incorporate these in our Discussion, for example, in relation to the DNMT3B over-expression study (now lines 327-334), and the explanation favoured by the reviewer (now lines 337-339).

Lines 327-334: "The protection of these domains is exemplified by a Dnmt3b overexpression mouse model that shows aberrant gain of methylation genome-wide and a marginal increase (<10%) across H3K27me3-marked domains (ref. 75). While DNMT3s have a strong affinity towards H3K36me2/3, there may be a weak affinity towards H3K27me3, such that the abundance of different DNMT3s and their interacting proteins could modulate chromatin localisation depending on the cellular context. A direct association of DNMTs with H3K27me3 regions in some contexts is supported by the observation that DNA methylation is dependent on the deposition of H3K27me3 at a subset of CGIs in extraembryonic ectoderm (ref.76) and cancer (ref. 77)."

Lines 337-339: "One possibility is that H3K36me2/3 acts as a sink for wild-type DNMT3A, and loss of affinity for this interaction would permit mutant protein to localise to bivalent chromatin exclusively, tipping the balance between DNMT3A and TETs at those sites."

6) p 13, line 310+311: recently several papers reported binding of PRC2 to CpG methylated DNA. This sentence should be updated.

>> As recommended, we have updated the statement to include these findings:
Lines 349-351: "The H3K27me3 that remains at the hypermethylated DMRs…or could be maintained by PRC2 variants that show affinity for methyl-CpG (ref.79)."

7) p. 13, line 312: There were effects observed in E7.5, but they were not properly analyzed. This sentence at least should be removed.

>> The original sentence was: "we only observed ectopic DNA methylation in the presence of the *Dnmt3a*^{D329A} allele postnatally", in which ectopic referred to hypermethylation, which was only observed from P1. To clarify, we have altered the sentence to "we only observed hypermethylation of DNA in the presence of the *Dnmt3a*^{D329A} allele postnatally" (lines 352-353). As noted in our response to point 3) above, we have extended the analysis and description of the effects on DNA methylation at E7.5.

8) p. 13, line 328-330: I do not agree with this sentence. See point 5.

>> As noted in response to point 5) above, we have included alternative explanations in the Discussion at the reviewer's suggestion, but believe it is valid to state our preferred model in that context of the Discussion, with some qualification added (lines 369-372).

Reviewer #2 (Remarks to the Author):

The revised manuscript is improved, and my previous questions were addressed to some extent, although a few responses are still not quite convincing.

For example, the linkages among several main findings of the paper are still lacking. The additional analysis comparing H3K27me3 and DNA hypermethylation in mutants provides helpful supporting evidence but is still a correlation rather than direct proof of Dnmt3a recruitment.

>> The observed increase in CpG methylation, and especially non-CpG methylation, over discrete regions of the genome is considered a direct measure that there has been ectopic recruitment of a *de novo* DNA methyltransferase, and is frequently utilised in the literature (Baubec et al. 2015 Nature; Guo et al. 2014 Nat Neurosci; Morselli et al. 2015 eLife). Unfortunately, there are substantial technical challenges in assaying the recruitment of DNMT3A by other means. Direct binding studies by ChIP-seq for DNMT3A are really not feasible, as it is widely recognised that the available antibodies are not sufficiently specific and therefore require tagged proteins (Baubec et al. 2015 Nature, Manzo et al. 2017 EMBO J). We have considered developing tagged proteins in a cell culture system for pull-down studies. However, our work *in vivo* strongly suggests that there are important contexts to the activity of the DNMT3A mutant, as ectopic methylation is accumulating most prominently in postnatal neural tissues. Important factors that would be difficult to recapitulate in a cell culture system would be the abundance of additional DNMTs and DNMT3A isoforms, a post-replicative state, and the presence of potentially unknown co-factors. Thus, assaying protein-protein interactions or chromatin recruitment of DNMT3A in the wrong cellular context will likely produce ambiguous results and would be insufficient to conclude what molecular mechanisms are at play in postnatal neuron development. Hence, based on current methodologies, we feel that CpG and non-CpG methylation provide the best direct evidence of DNMT3A recruitment.

Also this again does not fully exclude the possibility that this is because these regions are hypomethylated from the beginning.

>> We have provided what we believe to be strong evidence to show that hypomethylation itself was not sufficient to recruit the mutant DNMT3A, but that it was strongly dependent on the presence of H3K27me3. This is shown in detail in the ChIP-seq analysis in Fig. 6, where only hypomethylated domains with H3K27me3 enrichment are subject to DNA methylation gain. However, as raised by Reviewer 1 (point 5), we do agree there are alternative mechanisms that could explain this patterning and, as such we, have added discussion of alternative mechanisms in the Discussion.

Second, I am still not fully convinced that differentially expressed genes expressed at basal levels (FPKM < 1 in both WT and mutant) are functional. This is not impossible but certainly requires functional evidence. Statistics are helpful but are known to be mis-leading sometimes.

>> We have taken a statistically robust approach to the RNA-seq data analysis that is consistent with the standards in the literature (Anders et al. 2013 Nat Protoc; Blanco et al. 2016 Nature; Kumasaka et al. 2018 Nat Genet), and we also explained why this may well be the level of de-repression one might expect in a complex tissue in which only a sub-population of cells may be affected. In addition, we did not rely only on RNA-seq results, but also found overlap with DNA methylation and chromatin alterations, etc., finding multiple molecular changes to support the observed de-repression (for example, shown for specific genes in new Fig. 7).

Regarding functional evidence, it would not be feasible to obtain this in a realistic time-frame. In particular, because the growth phenotype could be complex, the whole-animal phenotype might depend on the cumulative effect of deregulation of multiple genes. One could envisage an experiment to test the effect of de-repression of a given *Hox* gene, for example. It might involve

seeking to rescue the phenotype by crossing our mutant with a knock-out for that gene. However, it would then be likely that the *Hox* gene KO would have a developmental phenotype that overwhelms the effect we are seeking to rescue, so the cross would be uninformative. We feel these experiments are outside the scope of the current manuscript.

Finally, it is not clear to me why the relationship between DNA hypermethylation and gene expression is a "subsidiary question", given the obvious linkage between DNA methylation and gene expression.

>> Our apologies for using the term 'subsidiary' in our previous response. It was not meant to suggest that the linkage between DNA methylation and gene expression is unimportant, rather that the focus of the study is the unexpected targeting of DNA methylation to H3K27me3-marked chromatin. The relationship between regions gaining DNA methylation and gene expression in our study may be complex, and some of the connections may be obscured by the analysis of complex tissues. As we describe in the manuscript, amongst the most de-repressed genes there are examples in which there is gain of methylation at their promoters (Fig. S8), but even at these, the immediate transcription start site remains hypomethylated relative to flanking sequences (Fig. S11 and specific examples in Fig. 7). At this time, we would favour an explanation that gain of DNA methylation across the gene has led to some displacement of PRC2 and change in promoter state from 'poised' bivalent H3K4me3/H3K27me3 to a potentially more active H3K4me3 state (as in Fig. 7a). This possibility is presented in the Discussion (lines 340-351) and has similarities with the targeting of DNMT3A to *HOX* loci by using a dCas9 fusion (ref. 78).

I understand some of these mechanistic experiments are challenging within the revision period. Yet the response letter appeared to be poorly written. The related conclusions above should be at least tuned down, before it can be considered for publication in NC.

>> As detailed in our response to Reviewer 1, we have provided alternative mechanisms in the Discussion that could also explain our observations. Furthermore, in discussing our results and interpretation of them, we have now highlighted areas that require further work for definitive evaluation of these potential mechanisms.

REVIEWERS' COMMENTS:

Reviewer #1 (Remarks to the Author):

This is a very strong paper, very good science, nicely preseneted and fully convincing. My comments were considered in an adequant manner. I have just two small points the authors may wish to consider in the final editing steps:

1) Regarding the Gain-of-function effect of D329A. I find it exciting that this mutation, which was designed to have lost K36me2/3 binding, has a clear loss-of-function effect at the molecular level (because K36me2/3 binding is disrupted). Still at the organismic level it causes a complex gain-of-function effect.

2) Regarding the statement in the discussion that DNA methylation inhibits PRC2 binding (line 347), the autors may wish to consider PMID: 29058709.

NCOMMS-18-37103A

REVIEWERS' COMMENTS:

Reviewer #1 (Remarks to the Author):

This is a very strong paper, very good science, nicely preseneted and fully convincing. My comments were considered in an adequant manner. I have just two small points the authors may wish to consider in the final editing steps:

1) Regarding the Gain-of-function effect of D329A. I find it exciting that this mutation, which was designed to have lost K36me2/3 binding, has a clear loss-of-function effect at the molecular level (because K36me2/3 binding is disrupted). Still at the organismic level it causes a complex gain-of-function effect.

> >> We are very pleased the reviewer found our study findings exciting, and we share his/her enthusiasm. We agree that it is useful to contrast the biochemical prediction that was the premise of the study with the complex,organismal phenotype that was observed. We think it is most appropriate to emphasise this outcome at the end of the summary paragraph in the Introduction, therefore, we have added the following sentence.
>
> "Thus, a mutation in DNMT3A predicted to abrogate an interaction with H3K36me2/3 causes a complex, gain-of-function phenotype *in vivo* associated with recruitment of DNMT3A towards H3K27me3-marked chromatin."

2) Regarding the statement in the discussion that DNA methylation inhibits PRC2 binding (line 347), the autors may wish to consider PMID: 29058709.

> >> We have now updated the statement and included the recommended reference (79).
> "This is supported by the observation that DNA methylation generally, albeit not always, inhibits PRC2 bining and activity *in vitro*[65,79] and can block the deposition of H3K27me3[66]."
